# Harnessing strong metal–support interactions via a reverse route

**DOI:** 10.1038/s41467-020-16674-y

**Published:** 2020-06-16

**Authors:** Peiwen Wu, Shuai Tan, Jisue Moon, Zihao Yan, Victor Fung, Na Li, Shi-Ze Yang, Yongqiang Cheng, Carter W. Abney, Zili Wu, Aditya Savara, Ayyoub M. Momen, De-en Jiang, Dong Su, Huaming Li, Wenshuai Zhu, Sheng Dai, Huiyuan Zhu

**Affiliations:** 1 Chemical Sciences Division, Oak Ridge National Laboratory, Oak Ridge, TN 37831 USA; 2 School of Chemistry & Chemical Engineering, Jiangsu University, Zhenjiang, 212013 China; 3 Department of Chemical Engineering, Virginia Polytechnic Institute and State University, Blacksburg, VA 24061 USA; 4 Department of Chemistry, University of California, Riverside, CA 92521 USA; 5 Center for Functional Nanomaterials, Brookhaven National Laboratory, Upton, NY 11973 USA; 6 Frontier Institute of Science and Technology, Xi’an Jiaotong University, Xi’an, 710054 China; 7 Neutron Scattering Division, Oak Ridge National Laboratory, Oak Ridge, TN 37831 USA; 8 Energy and Transportation Science Division, Oak Ridge National Laboratory, Oak Ridge, TN 37831 USA; 9 Joint Institute for Advanced Materials, University of Tennessee, Knoxville, TN 37996 USA

**Keywords:** Catalysis, Materials for energy and catalysis, Nanoscale materials, Characterization and analytical techniques

## Abstract

Engineering strong metal–support interactions (SMSI) is an effective strategy for tuning structures and performances of supported metal catalysts but induces poor exposure of active sites. Here, we demonstrate a strong metal–support interaction via a reverse route (SMSIR) by starting from the final morphology of SMSI (fully-encapsulated core–shell structure) to obtain the intermediate state with desirable exposure of metal sites. Using core–shell nanoparticles (NPs) as a building block, the Pd–FeO_x_ NPs are transformed into a porous yolk–shell structure along with the formation of SMSIR upon treatment under a reductive atmosphere. The final structure, denoted as Pd–Fe_3_O_4_–H, exhibits excellent catalytic performance in semi-hydrogenation of acetylene with 100% conversion and 85.1% selectivity to ethylene at 80 °C. Detailed electron microscopic and spectroscopic experiments coupled with computational modeling demonstrate that the compelling performance stems from the SMSIR, favoring the formation of surface hydrogen on Pd instead of hydride.

## Abstract (web summary)

Strong metal–support interactions (SMSI) are effective in tuning the structures and catalytic performances of catalysts but limited by the poor exposure of active sites. Here, the authors develop a strategy to engineer SMSI via a reverse route, which is in favor of metal site exposure while embracing the SMSI.

## Introduction

Supported metal catalysts have long been recognized as the most important group of heterogeneous catalysts for fundamental investigations and modern chemical industries^1–4^. Conventionally, these catalysts are synthesized by anchoring the active metal nanoparticles (NPs) onto certain high-surface-area supports to increase the dispersion of catalytically active sites and stabilize the metal against leaching^5–7^. Subsequently, the metal–support interface is constructed. Such an interface provides synergistic properties to regulate catalysis by modifying the electronic (charge transfer between the metal sites and the support) and/or geometric (decoration or coverage of metal sites by the support) parameters, and also by modulating the reaction pathways, e.g., lattice oxygen in oxide supports may directly participate in catalytic reactions^7^; multicomponent interfaces can enable tandem reaction pathways that do not exist on single-component active sites^8,9^.

As a classic prototype in metal–support interactions, the strong metal–support interaction (SMSI) has been defined as the encapsulation of NPs, usually group VIII metals, by partially reduced oxide supports during high-temperature hydrogen (H_2_) treatment^10,11^. Since the very first discovery of SMSI by Tauster et al.^12–14^, SMSI has been widely exploited to tune catalytic performances of group VIII NPs by engineering geometric and/or electronic structures of these metal sites. For example, the adsorption of H_2_ or CO on Pd was extremely suppressed upon the formation of SMSI (refs. ^13,15^), suggesting that the active metal sites were largely covered by support, which altered the geometric ensembles and improved the thermal stability of Pd catalysts. Meanwhile, because the reducible oxide support, e.g., TiO_2_, Co_3_O_4_, CeO_2_, and Nb_2_O_5_, is partially reduced to the structure with a nonstoichiometric oxygen concentration during the reductive annealing, electron transfer between metal NPs and oxide supports was detected^16–19^. Under extreme conditions, the formation of intermetallic structure of the supported metal and metal cations in the supporting oxide was observed^20,21^.

Despite these fascinating interfacial properties in SMSI, the formation of SMSI is restricted to specific combinations of elements, i.e., group VIII metals with high surface energy and transition metal oxides with low surface energy. Consequently, it is extremely challenging for some metals, e.g., Au, to manifest SMSI due to their low work function and surface energy^15,17,22^. Efforts have been devoted in hope of expanding upon the conventional SMSI. One critical element in this pursuit is switching the high-temperature treatment in H_2_ into other conditions and thereby changes the mechanistic pathways for the formation of SMSI. For example, Wang et al. reported SMSI formation between Au NPs and TiO_2_ induced by melamine under an oxidative atmosphere. With the formation of SMSI, the Au NPs were encapsulated by a permeable TiO_x_ thin layer, making the Au NPs ultrastable at 800 °C (ref. ^23^). Xiao et al. reported a wet chemistry approach to construct SMSI in aqueous solution at room temperature, which was realized by engineering redox interactions between metals and supports. This strategy was applicable to Au, Pt, Pd, and Rh (ref. ^15^). Christopher et al. developed a strongly bounded-adsorbate-mediated strategy to construct SMSI between Rh and TiO_2_ through high-temperature treatment in the mixture of CO_2_ and H_2_ (ref. ^24^). Zhang et al. engineered the SMSI between Au NPs and hydroxyapatite by treating the Au NP–hydroxyapatite composite in the air at high temperatures^17^. Although progress has been made in expanding the boundaries of SMSI, one inevitable issue associated with the conventional SMSI is that upon high-temperature treatment the encapsulation process immediately and uncontrollably takes place, resulting in limited exposure of active sites^25^. In the ideal scenario, the oxide coverage on the metal surface needs to be thin and permeable to small molecules, while still fully encapsulating metal NPs to prevent the dissolution, disintegration, and aggregation of active sites during catalysis.

**Fig. 1:**
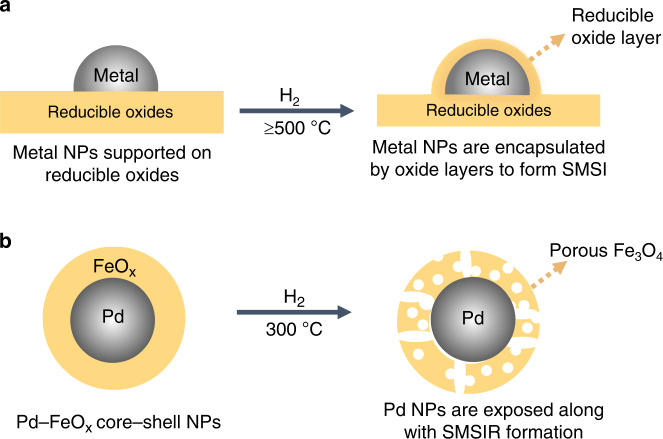
Schematic illustration of the formation of strong interactions. **a** The conventional SMSI formation process. **b** The SMSIR formation process in this work.

We recently reported that voids and cavity space can be developed in metal–metal oxide core–shell NPs in response to H_2_ treatment at 200 °C (ref. ^26^). This observation combined with the current issues in conventional SMSI motivated us to develop alternative routes to metal–support interactions. Here, we denote this type of structural rearrangement as the strong metal–support interaction via a reverse route (SMSIR). Specifically, we start from the final morphology of SMSI (full encapsulation) and end in the intermediate state with partial exposure of metal sites (Fig. 1). As a proof of concept, we demonstrate that the core–shell Pd–FeO_x_ NPs can be restructured into a porous yolk–shell structure after optimized reductive annealing (Pd–Fe_3_O_4_–H). Characterizations reveal that Pd atoms gradually migrate into the Fe_3_O_4_ lattice and electron is partially transferred from Pd to Fe_3_O_4_. The Pd–Fe_3_O_4_–H shows 100% conversion and 85.1% selectivity in the acetylene (C_2_H_2_) semi-hydrogenation at atmospheric pressure and a mild reaction temperature of 80 °C. Further investigations demonstrate that the Pd–Fe_3_O_4_–H engineered by the SMSIR alleviates the strong H_2_ adsorption on Pd sites, in favor of the formation of surface hydrogen (surface-H) instead of hydride during the hydrogenation of C_2_H_2_ to C_2_H_4_. Our results on the scenario of engineering SMSIR can help to circumvent the current limits in metal–support interfaces, expanding the boundaries of conventional SMSI, and providing opportunities to rationally maneuver structure-dependent catalytic outcomes.

## Results

### Synthesis and characterization

Details of the material synthesis can be found in the “Methods” section. Briefly, the Pd NPs with a size of 5.5 ± 0.5 nm (Supplementary Fig. 1) were prepared by the reduction of palladium (II) acetylacetonate (Pd(acac)_2_) in oleylamine (OAM) as modified from a previous report^27^. The core–shell Pd–FeO_x_ NPs were obtained by a seed-mediated growth method with the pre-made Pd NPs as the seeds and iron (III) acetylacetonate, as the iron precursor that nucleated on Pd surface, forming an iron oxide shell. The pristine core–shell sample was denoted as Pd-FeO_x_ NPs (Supplementary Figs. 2 and 3). The SMSIR was constructed by treating the Pd–FeO_x_ NPs at 300 °C in a gas mixture of H_2_ and argon (Ar; 4 vol.% of H_2_), and the sample was named as Pd–Fe_3_O_4_–H. As a comparison, the Pd–FeO_x_ NPs were treated in the air at 300 °C to obtain the structure without SMSIR (Pd–Fe_3_O_4_–A).

X-ray diffraction (XRD) was performed to determine the crystal structures of the samples. As shown in the XRD patterns of pristine Pd–FeO_x_ and Pd–Fe_3_O_4_–A (Supplementary Fig. 4), characteristic peaks at 2*θ* = 40.1° with very low intensities were detected, which can be assigned to the (111) peak of face-centered cubic (*fcc*) Pd. No additional peaks in the XRD patterns can be found, indicating the amorphous nature of iron oxide shell in both pristine Pd–FeO_x_ and Pd–Fe_3_O_4_–A. On the contrary, in the XRD pattern of Pd–Fe_3_O_4_–H, the intensity of Pd (111) peak increases remarkably, and a series of characteristic peaks at 2*θ* = 30.6°, 35.9°, 43.5°, 53.9°, 57.3°, 63.0°, and 74.3° are clearly observed, which can be assigned to (220), (311), (400), (422), (511), (440), and (533) lattices of *γ*-Fe_3_O_4_. The XRD characterization indicates that annealing in the reductive atmosphere may facilitate the spatial redistribution of grains in the oxide shell, and promotes the crystallization of Pd and iron oxides, consistent with our previous report^26^.

**Fig. 2:**
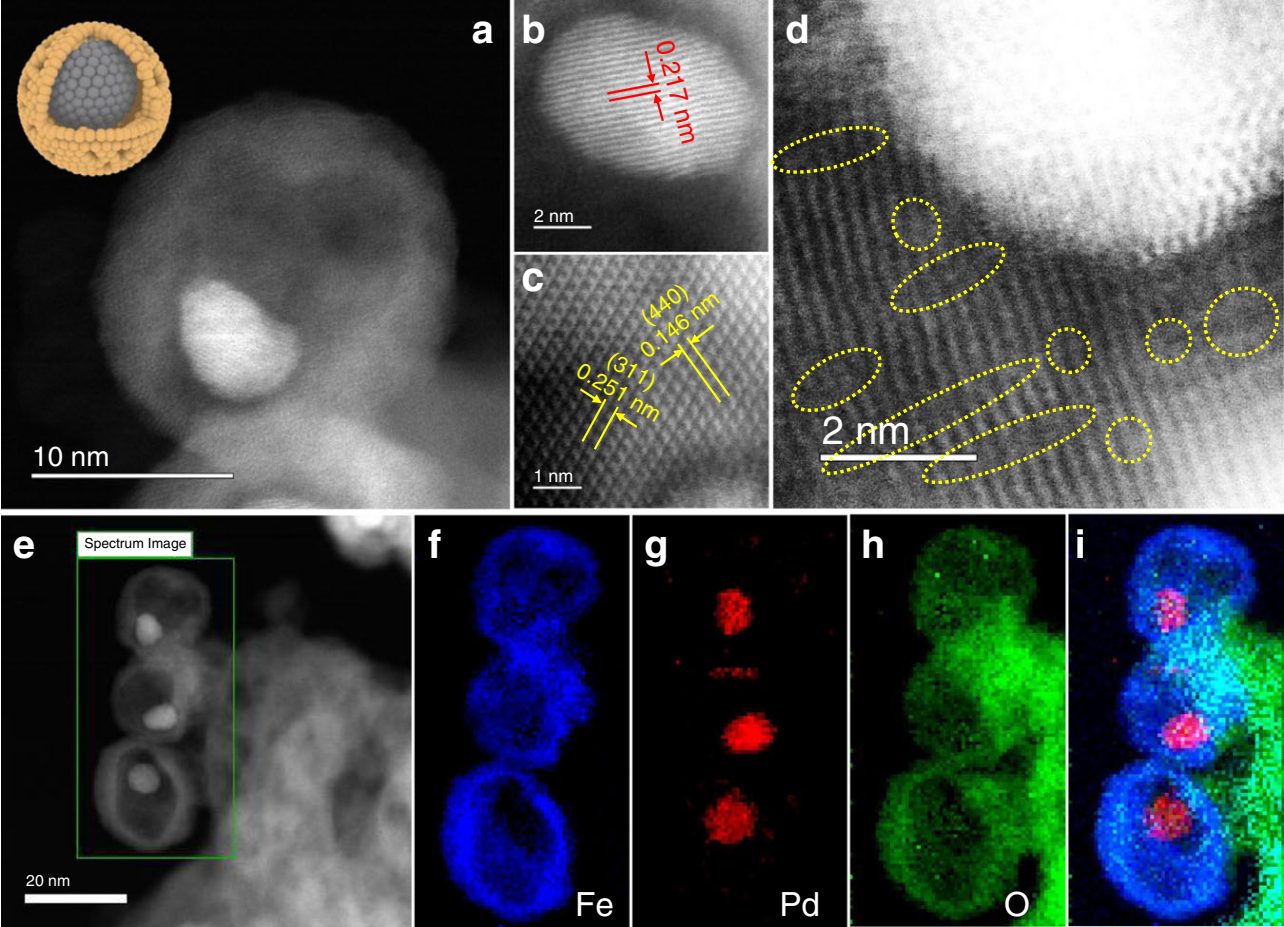
STEM characterization of the Pd–Fe_3_O_4_–H sample. **a** HAADF-STEM image of Pd–Fe_3_O_4_–H and schematic illustration of the structure (the yellow shell stands for Fe_3_O_4_, and grey core stands for Pd); **b** HR-STEM images of Pd core, **c** Fe_3_O_4_ shell, and **d** Pd–Fe_3_O_4_–H (voids in oxide shell are marked in yellow circles); **e** STEM image of Pd–Fe_3_O_4_–H at lower magnification; **f**–**i** corresponding EELS elemental mappings of the selected section in **e**; **f** Fe, **g** Pd, **h** O, and **i** overlapped figure.

The aberration-corrected high-angle annular dark-field scanning transmission electron microscopy (HAADF-STEM) images of the Pd–Fe_3_O_4_–H in Fig. 2a show that the core–shell structure of pristine Pd–FeO_x_ NPs evolved into a unique porous yolk–shell structure after reductive annealing at 300 °C. Magnified HR-STEM images of Pd–Fe_3_O_4_–H in Fig. 2b, c demonstrate a lattice parameter of 0.217 nm in the core, corresponding to the (111) plane of Pd, and lattice parameters of 0.251 and 0.146 nm in the shell, corresponding to the (311) and (440) planes of Fe_3_O_4_. More interestingly, the magnified HR-STEM image in Fig. 2d shows that there are abundant voids, i.e., lattice vacancies, in the Fe_3_O_4_ shells (marked in yellow circles). To analyze the pore distribution, the pore sizes were determined by averaging pore sizes in multiple HR-STEM images (Supplementary Fig. 5). The majority of these pores on the Fe_3_O_4_ shell are micropores with an average pore size of 0.73 nm. Furthermore, electron energy loss spectroscopy (EELS) mapping of Pd–Fe_3_O_4_-H in Fig. 2e–i depict a yolk–shell-like structure of Pd yolk and Fe_3_O_4_ shell with numerous voids. In contrast, for Pd–Fe_3_O_4_–A, no significant voids were detected in the Fe_3_O_4_ shells and the intact core–shell structure was retained (Supplementary Fig. 6). It is known that the reducible metal oxides can be partially reduced after high-temperature treatment in H_2_ (ref. ^28^). H_2_ reacts with these oxides to produce water and generate oxygen vacancies in the oxide matrix. This process can be further facilitated by platinum-group metal NPs supported on those oxides through a H_2_ spillover process^29,30^. In the meantime, the crystallization of oxide shell could promote the rearrangement of atoms, alter the distribution of oxide grains to expand the generated oxygen vacancies, and finally develop voids and cavity space in the structure.

### XAFS characterization and simulations

**Fig. 3:**
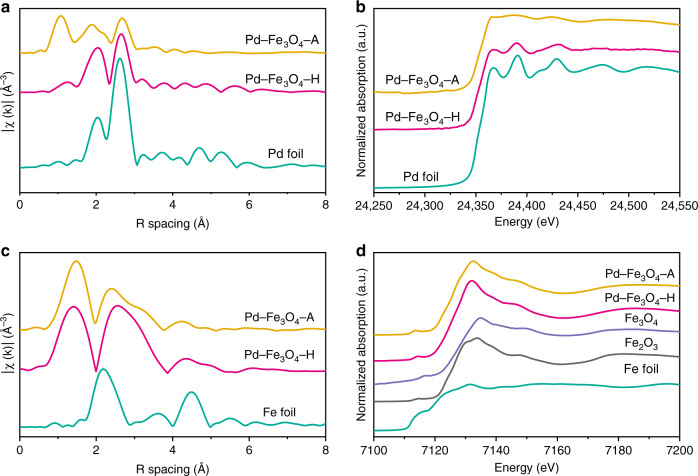
XAFS characterization of prepared samples and reference samples. **a** Pd K-edge EXAFS; **b** Pd K-edge XANES; **c** Fe K-edge EXAFS; and **d** Fe K-edge XANES. Source data are provided as a Source data file.

To understand the coordination environments of Pd–Fe_3_O_4_ structures, X-ray absorption near-edge spectroscopy (XANES) and extended X-ray absorption fine structure (EXAFS) were performed (Fig. 3, Supplementary Tables 1 and 2). The chemical states of Pd in Pd–Fe_3_O_4_–H and Pd–Fe_3_O_4_–A samples were investigated in Pd K-edge EXAFS and XANES, and Pd foil was employed as a reference (Fig. 3a, b, Supplementary Table 1). The Pd in Pd–Fe_3_O_4_–H mainly exists in the form of metallic Pd^0^, while in Pd–Fe_3_O_4_–A, Pd demonstrates an oxidized feature to some extent. To determine the chemical states and structures of iron oxide shells, the Fe K-edge EXAFS (Fig. 3c), corresponding fitting (Supplementary Table 2), the Fe K-edge XANES (Fig. 3d), and the Fe K-edge first derivative XANES (Supplementary Fig. 7) were collected and analyzed. Compared with the Fe_3_O_4_ and Fe_2_O_3_ references, the Fe K-edge XANES and the Fe K-edge first derivative XANES indicated that the oxide shell in Pd–Fe_3_O_4_–H was similar to Fe_3_O_4_, while the oxide shell in Pd–Fe_3_O_4_–A possessed a partially oxidized Fe_3_O_4_ feature (Fig. 3d, Supplementary Fig. 7).

**Fig. 4:**
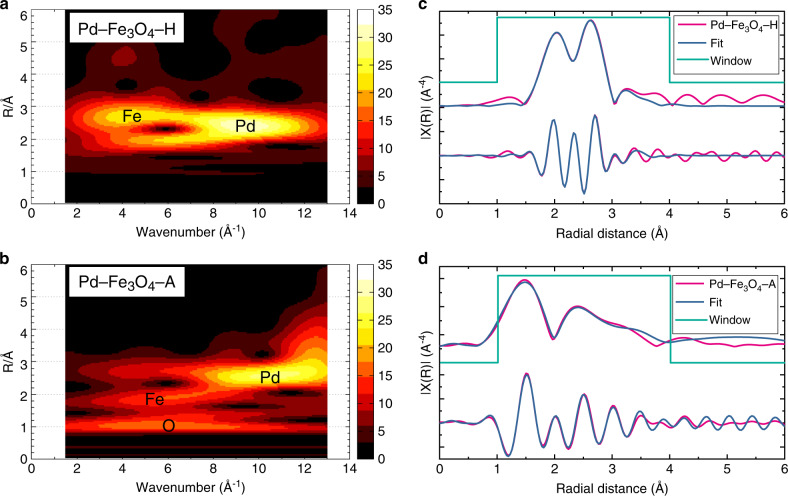
Pd K-edge WT EXAFS and EXAFS fitting using DFT-optimized results. **a** Pd K-edge WT EXAFS of Pd–Fe_3_O_4_–H; **b** WT EXAFS of Pd–Fe_3_O_4_–A; **c** EXAFS curve fitting on DFT-optimized Pd–Fe_3_O_4_–H model; and **d** EXAFS curve fitting on DFT-optimized Pd–Fe_3_O_4_–A model. Source data are provided as a Source data file.

Because Pd–Pd and Pd–Fe bond lengths are similar, it is hard to visualize the difference between these two bonds with Fourier transform results. In this regard, the wavelet transform (WT) EXAFS as a powerful technique was employed to distinguish these two bonds in our samples. It can be clearly seen from Fig. 4a, b that compared with the standard WT EXAFS images for Pd–Fe, Pd–O, Pd–Pd, and Pd foil (Supplementary Fig. 8), the Pd in Pd–Fe_3_O–H remains to be the metallic Pd^0^ state and Fe–Pd bond emerges (Fig. 4a). In contrast, for the Pd–Fe_3_O_4_–A sample (Fig. 4b), the result demonstrates an oxidized feature with the formation of the Pd–O bond, indicating that the Pd may be slightly oxidized by air, consistent with our EXAFS and XANES results in Fig. 3.

Density functional theory (DFT) simulations combined with the EXAFS curve fitting were carried out to provide more insight into the crystal structure of iron oxide, and the interactions between Pd and Fe_3_O_4_. First, a series of models including a Pd cluster atop the surfaces of Fe_2_O_3_ and Fe_3_O_4_, and a Pd cluster in oxygen vacancy of Fe_2_O_3_, and Fe_3_O_4_ surfaces were constructed and optimized by DFT in Supplementary Fig. 9, and the corresponding FEFF calculated scattering paths were also presented (Supplementary Tables 3–5). Then, the EXAFS curve fitting on the DFT-optimized structures (Supplementary Figs. 10 and 11, Supplementary Tables 6 and 7) of both Pd K-edge EXAFS and Fe K-edge EXAFS were obtained. It can be concluded from the results that the best-fitted structure of Pd–Fe_3_O_4_–H is that the Pd atoms intercalate into the Fe_3_O_4_ matrix (Fig. 4c, detailed optimizing process see Supplementary Fig. 10), indicating that the Pd enters into the Fe_3_O_4_ lattice, substituting an oxygen vacancy and tends to form the Fe–Pd bond with Fe in Fe_3_O_4_. This observation suggests that there exist strong interactions between Pd and Fe_3_O_4_ in Pd–Fe_3_O_4_–H. In contrast, the Pd–Fe_3_O_4_–A demonstrated a good match to the local geometry of Pd atoms situated on the surface of Fe_3_O_4_ (Fig. 4d, detailed optimizing process see Supplementary Fig. 11). This result may shed some light on the formation mechanism of this unique porous yolk–shell structure. Evidently, the reaction between H_2_ molecules and O atoms in the Fe_3_O_4_ could generate oxygen vacancies in the structure upon evaporating the produced water. Meanwhile, the crystallization of the oxide shell and the new Fe–Pd bond formation could promote the rearrangement of oxide lattice and the mobility of Pd atoms, expanding the atom vacancies and developing cavity space in the structure.

### XPS and DRIFTS investigations

To investigate the charge transfer with the formation of SMSIR, X-ray photoelectron spectroscopy (XPS) and CO diffuse reflectance infrared Fourier transform spectroscopy (DRIFTS) were carried out and shown in Supplementary Figs. 12–14. In the high-resolution Pd 3*d* XPS of Pd–FeO_x_ NPs, only a Pd 3*d*_5/2_ peak at 335.4 eV, being assigned to metallic Pd, was found^31,32^. When the Pd–FeO_x_ NPs were treated in air at 300 °C, an additional Pd 3*d*_5/2_ peak at 336.8 eV assigned to PdO, emerged in Supplementary Fig. 12. This observation is consistent with our XAFS results that the Pd in Pd–Fe_3_O_4_–A possesses an oxidized feature to some extent. Furthermore, a Pd 3*d*_5/2_ shoulder peak at 336.2 eV in the high-resolution Pd 3*d* XPS of Pd–Fe_3_O_4_–H was detected and assigned to the positively charged Pd (Pd^δ+^)^33^, which is originated from intercalation of Pd into Fe_3_O_4_ matrix, leading to the strong interactions between Pd and Fe to form Pd–Fe bond^34^, in accordance with the XAFS results. Meanwhile, in the high-resolution Fe 2*p* XPS of Pd–Fe_3_O_4_–H (Supplementary Fig. 13), the peaks of Fe 2*p*_1/2_ and Fe 2*p*_3/2_ downshifted comparing with that of Pd–Fe_3_O_4_–A, further confirming that the charge transfers from Pd to Fe_3_O_4_ in Pd–Fe_3_O_4_–H. The XPS results indicate the formation of strong interactions and partial electron transfer between Pd and Fe_3_O_4_. The CO DRIFTS was further carried out. During the test, we found that the CO adsorption peak was weak. We, therefore, subtracted the pure gas-phase signal from each data set. As shown in Supplementary Fig. 14, a peak at ~2153 cm^−1^ was detected both in the CO DRIFTS of Pd–Fe_3_O_4_–H and Pd–Fe_3_O_4_–A, which is assigned to Fe^3+^–CO (ref. ^35^). Due to the core–shell morphology of Pd–Fe_3_O_4_–A where Pd is fully encapsulated by Fe_3_O_4_, no obvious peak was detected in the CO DRIFTS of Pd–Fe_3_O_4_–A. In contrast, a very weak peak at 2102 cm^−1^ can be seen in the CO DRIFTS of Pd–Fe_3_O_4_–H, which is assigned to the linear CO adsorption on metallic Pd (ref. ^36^). More interestingly, an additional peak at 2134 cm^−1^ can be found. Compared with the linear CO adsorption on metallic Pd, this blueshifted peak is assigned to linear CO adsorption on positively charged Pd (CO–Pd^δ+^)^37^. Combined with the XPS analysis, this peak may be attributed to the linear CO adsorption on Pd^δ+^ in the newly emerged Pd–Fe bond of Pd–Fe_3_O_4_–H.

The Pd–Fe_3_O_4_–H sample was further re-treated in air at 300 °C to obtain the Pd–Fe_3_O_4_–Re sample, and characterized by TEM, CO DRIFTS, and XPS to determine the reversibility of SMSIR. As shown in the TEM image of the Pd–Fe_3_O_4_–Re (Supplementary Fig. 15), the sample still possesses a yolk–shell structure, but the voids are smaller than those of Pd–Fe_3_O_4_–H. The XPS of Pd–Fe_3_O_4_–Re (Supplementary Fig. 16) demonstrates three Pd states of metallic Pd, Pd^δ+^ in Pd–Fe bond, and PdO. The intensity of Pd^δ+^ peak is lower than that of Pd–Fe_3_O_4_–H (Supplementary Fig. 12), indicating the decrease of Pd^δ+^ concentration. The CO DRIFTS of Pd–Fe_3_O_4_–Re in Supplementary Fig. 17 shows that the intensity of CO–Pd^δ+^ became weaker than that in the CO DRIFTS of Pd–Fe_3_O_4_–H in Supplementary Fig. 14, suggesting the CO DRIFTS spectral feature is an intermediate state between Pd–Fe_3_O_4_–H and Pd–Fe_3_O_4_–A. The analysis of TEM, CO DRIFTS, and XPS together suggests that the SMSIR in this work is partially reversible.

### Catalytic performance

The semi-hydrogenation of C_2_H_2_ to C_2_H_4_ is an important reaction in industrial purification of the C_2_H_4_ stream produced from naphtha cracking. Pd-based catalysts are mostly used for this reaction with a consensus that the selectivity is sensitive to the structure of the catalyst^38^. H_2_ molecules that are weakly adsorbed onto the Pd surface to form surface-H and C_2_H_2_ molecules that are strongly adsorbed can lead to the production of C_2_H_4_, while the formation of hydride usually results in the total hydrogenation to ethane (C_2_H_6_). The adsorption of H_2_ and C_2_H_2_ strongly relies on the structure of Pd catalysts. Herein, the hydrogenation of C_2_H_2_ was systematically investigated over the prepared catalysts to correlate their structural properties with the catalytic outcomes.

**Fig. 5:**
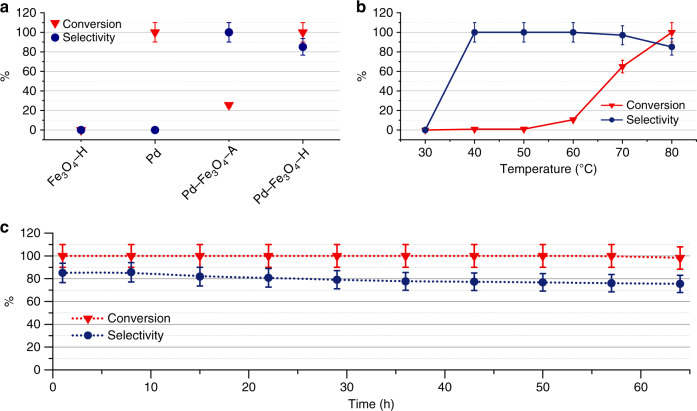
Catalytic performances of the prepared samples for the semi-hydrogenation of C_2_H_2_. **a** The comparison of catalytic performance of different systems; **b** light-off curves of the Pd–Fe_3_O_4_–H catalyst; **c** stability of Pd–Fe_3_O_4_–H under high conversion. Reaction conditions: *m* (catal.) = 15 mg; *v* (gas) = 50 sccm (0.6 sccm C_2_H_2_, 3 sccm H_2_, 46.4 sccm He). The reaction temperature in **a** and **c** is 80 °C. Error bars represent the instrumental error (±10 %). Source data are provided as a Source data file.

As shown in Fig. 5a, Pd NPs totally converted C_2_H_2_ to C_2_H_6_ without any selectivity toward C_2_H_4_ at 80 °C, while Fe_3_O_4_ NPs treated at 300 °C in a gas mixture of H_2_ and Ar (4 vol.% of H_2_; Fe_3_O_4_–H) barely demonstrated any catalytic activity for the hydrogenation of C_2_H_2_. The selectivity of C_2_H_2_ to C_2_H_4_ over the Pd–Fe_3_O_4_–A was 100%, but the conversion was only 25.6%. This observation can be mainly attributed to the core–shell structure of the Pd–Fe_3_O_4_–A that only exposes limited active Pd sites through the amorphous oxide shell, restricting the adsorption of the reactants. When the Pd–Fe_3_O_4_–H was employed in the semi-hydrogenation of C_2_H_2_ at 80 °C, the conversion was 100% and the selectivity was as high as 85.1%. The light-off curves of Pd–Fe_3_O_4_–H (Fig. 5b) demonstrate that the conversion of C_2_H_2_ increases with the increment of reaction temperature, while the selectivity toward C_2_H_4_ shows the opposite trend. To comprehensively compare the catalytic performance between the Pd–Fe_3_O_4_–H catalyst and previously reported values, the turnover frequency (TOF) was calculated based on the dispersion of Pd (obtained from H_2_-pulse chemisorption in Supplementary Table 8). The TOF of Pd–Fe_3_O_4_–H was 6.46 s^−1^, ~100-fold higher than those of a series of state-of-the-art single-atom catalysts at 80 °C (Supplementary Fig. 18), indicating that the Pd–Fe_3_O_4_–H demonstrated compelling catalytic performance for semi-hydrogenation of C_2_H_2_. Stability tests of the Pd–Fe_3_O_4_–H catalyst were further carried out under both the high and low conversion rates (Fig. 5c, Supplementary Fig. 19). Both results show that the Pd–Fe_3_O_4_–H catalyst was remarkably stable in the semi-hydrogenation of C_2_H_2_ to C_2_H_4_, which could be originated from the SMSIR between Pd and Fe_3_O_4_.

The formation of hydride in Pd-based catalysts is temperature-sensitive, and it dominates the total hydrogenation of C_2_H_2_ (refs. ^38,39^). Hence, the dispersion of Pd is determined by H_2_-pulse chemisorption (Supplementary Table 8) at various temperatures to examine the formation of hydride. For the reference Pd NPs (commercial 5 wt.% Pd/Al_2_O_3_), the dispersion was determined to be 7.6% at −130 °C (cold bath of isopentane and liquid N_2_). The corresponding particle size was calculated to be 14.8 nm. However, H_2_ uptake on Pd NPs increased significantly at 35 °C, and the estimated particle size decreased to 1.6 nm. This discrepancy can be attributed to the substantial formation of hydride on Pd NPs at higher temperature that interferes with the estimation of particle size^40^. In contrast, the Pd–Fe_3_O_4_–H sample demonstrated a dispersion of 26.7 and 24.4% at −130 and 35 °C, respectively. The corresponding particle sizes were calculated to be 4.2 and 4.6 nm, in agreement with the Pd core size from STEM investigations (Fig. 2). These observations indicate that the formation of hydride may be effectively inhibited in our Pd–Fe_3_O_4_–H catalyst with SMSIR, leading to a superior selectivity toward semi-hydrogenated products in the catalytic investigations.

### Control experiments

A series of control samples obtained at different treating temperatures (T200, T300, and T400) were prepared (see “Methods” section) to determine the optimized condition for the formation of SMSIR. Here, the T300 stands for the Pd–Fe_3_O_4_–H sample with SMSIR. The structures and compositions of all catalysts are characterized by TEM, STEM (Supplementary Figs. 20 and 21), EXAFS, XANES, EXAFS curve fitting on DFT-optimized model (Supplementary Figs. 22–29, Supplementary Tables 9–11), and XRD patterns (Supplementary Fig. 30). The corresponding structures are summarized here: in the pristine core–shell Pd–FeO_x_ sample, the core and shell were metallic Pd^0^ and amorphous Fe_3_O_4_. When the sample was treated in the air at high temperature (Pd–Fe_3_O_4_–A), the core–shell structures maintained with no obvious formation of voids. When the Pd–FeO_x_ NPs sample was treated in H_2_ at different temperatures, the core and shell crystallized into Pd^0^ and Fe_3_O_4_, respectively. As a result, the T200 demonstrated a core–shell structure with fewer voids, T300 (Pd–Fe_3_O_4_–H) embraced a yolk–shell-like structure with numerous voids, and the T400 showed a heterostructure of Fe_3_O_4_ islands on Pd NPs. Especially, it can be seen from the EXAFS and corresponding fitting results of T300, i.e., Pd–Fe_3_O_4_–H, (Fig. 3c, Supplementary Table 2) that compared with the sample obtained at lower annealing temperatures (T200 sample; Supplementary Fig. 24, Supplementary Table 10), the Fe–O coordination number decreased but Fe–Fe coordination number remained stable in Pd–Fe_3_O_4_–H. This result further suggests that in Pd–Fe_3_O_4_–H, Pd may substitute the oxygen in iron oxide and form a new Fe–Pd bond, indicating the formation of strong interactions between Pd NPs and Fe_3_O_4_ shell. In addition, Pd–FeO_x_ NPs with different shell thicknesses (STs) were also prepared. With the increment of STs, the samples were denoted as ST1 NPs, ST2 NPs (i.e., Pd–FeO_x_ NPs), and ST3 NPs, and the NPs were further loaded onto *γ*-Al_2_O_3_ to obtain ST1, ST2 (i.e. Pd–Fe_3_O_4_–H), and ST3 (for characterizations see Supplementary Figs. 31–34). To help understand the structures of all prepared samples, a schematic diagram was presented in Supplementary Figs. 35 and 36.

To highlight the role of SMSIR in tuning the conversion and selectivity of C_2_H_2_ semi-hydrogenation, both sets of control samples were employed in the C_2_H_2_ semi-hydrogenation reaction. As shown in Supplementary Figs. 37 and 38, the Pd–Fe_3_O_4_–H, i.e., T300 and ST2, demonstrates the best catalytic performance. This result further reveals that the optimized ST and treating condition are essential to the formation of SMSIR for the promoted semi-hydrogenation of C_2_H_2_ to C_2_H_4_. Based on the structures of the catalysts, the different catalytic outcomes can be attributed to the following factors: (1) regarding the effect of annealing temperatures, all samples possess a similar Pd size, indicating that the difference of catalytic performance is not originated from the difference of particle sizes (Supplementary Fig. 39). In the T200 sample, there are fewer voids in the oxide shell and the T200 remains to be a core–shell structure, resulting in poor exposure of Pd active sites with limited activity. In the case of T400 sample, the core–shell structure is completely destroyed, and therefore the formation of hydrides turns to be favorable because of the loss of core–shell structural confinement; (2) for the effect of ST, a thicker shell in ST3 makes the Pd active sites less exposed. However, when the shell becomes too thin as the case in ST1, the structure cannot maintain a fully-encapsulated state, but rather more like a heterostructure with some iron oxide islands on Pd NPs. Consequently, Pd domains tend to form hydrides due to the lack of core–shell structural confinement effect.

### Reaction mechanism

The reaction kinetics were further explored to understand the underlying mechanisms. As shown in Supplementary Fig. 40, the reaction order over C_2_H_2_ is calculated to be −1 (up to 2.5% atm partial pressure), roughly in agreement with Monnier’s work with ~−0.7 reaction order^41^, indicating the strong adsorption of C_2_H_2_ on the surface of Pd in the Pd–Fe_3_O_4_–H catalyst. There exists a debate regarding the H_2_ reaction order. In general, the reaction order varies from ~0.5 (refs. ^42,43^), ~1 (refs. ^44,45^), and up to ~1.6 (ref. ^46^). In our work, we found the reaction order of H_2_ to be ~2 (up to 10% atm partial pressure). Such a positive dependence on H_2_ partial pressure indicates a much weaker H_2_ adsorption than previous studies. The temperature dependence of the Pd–Fe_3_O_4_–H sample was investigated at 1.2%/6% atm partial pressure of C_2_H_2_/H_2_ (Supplementary Fig. 41). The apparent activation energy was found to be ~52.7 kJ mol^−1^, in good agreement with Monnier’s 12.1 kcal mol^−1^ (ref. ^41^) and Zhang’s 52 kJ mol^−1^ (ref. ^45^).

**Fig. 6:**
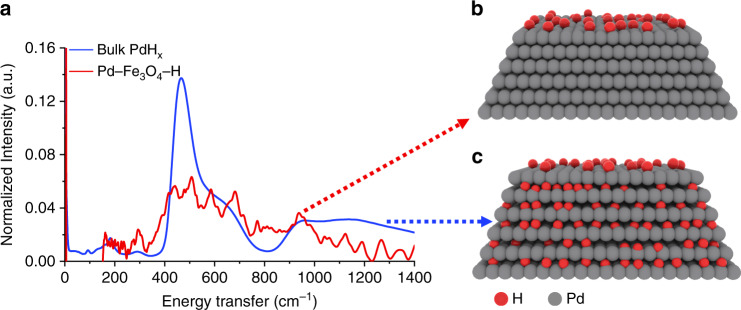
Determination of reaction intermediate species over Pd–Fe_3_O_4_–H and bulk PdH_x_. **a** INS spectra of Pd–Fe_3_O_4_–H and bulk PdH_x_; **b** schematic diagram of surface-H formed in Pd–Fe_3_O_4_–H; and **c** schematic diagram of hydride formed in bulk PdH_x_. Source data are provided as a Source data file.

The inelastic neutron scattering (INS) spectra of H_2_ adsorption on Pd–Fe_3_O_4_–H and bulk Pd were presented in Fig. 6. The signal of H_2_-sorption behavior in Pd–Fe_3_O_4_–H is totally different from that in bulk PdH_x_. In bulk PdH_x_ sample, an evident signal of hydride was detected, while in Pd–Fe_3_O_4_–H, the signal of hydride was very weak^47,48^. The profile of the peak at 500 cm^−1^ reflects the status of the hydride. Specifically, the sharp peak followed by a shoulder as seen in bulk PdH_x_ is due to certain dispersion relation of optical phonons in 3D space, which results in this particular distribution of phonon states. When hydride is only formed at or near the surface, the 3D network is lacking, leading to the broad bump in the spectrum of our Pd–Fe_3_O_4_–H sample. The result indicates that only surface-H formed during the reaction process, consistent with our H_2_-chemisorption results.

## Discussion

In this work, we reported a strategy to engineer the SMSI between Pd and Fe_3_O_4_ by using core–shell NPs as a building block through a reverse process of the formation of conventional SMSI, denoted as SMSIR. With the formation of SMSIR, the core–shell Pd–FeO_x_ NPs was restructured into a unique porous yolk–shell structured Pd–Fe_3_O_4_–H, in favor of the exposure of Pd active sites. The Pd–Fe_3_O_4_–H with SMSIR demonstrated excellent catalytic performance in semi-hydrogenation of C_2_H_2_ to C_2_H_4_ with 100% conversion, 85.1% selectivity, and a high TOF of 6.46 s^−1^ at the reaction temperature as low as 80 °C. XAFS investigations along with DFT simulations verified that the Pd atoms intercalate into the Fe_3_O_4_ matrix and form strong interactions. The electron transfer was probed by CO DRIFTS and XPS, suggesting that with the formation of SMSIR, electrons partially transfers from Pd to Fe_3_O_4_ shell. The optimized ST of Pd–FeO_x_ NPs and annealing temperature were found to be essential to the formation of SMSIR. Detailed mechanistic investigations indicated that the SMSIR in Pd–Fe_3_O_4_–H alleviates the strong chemisorption of H_2_ on Pd sites, prevents the formation of hydride, and consequently leads to a superior selectivity toward C_2_H_4_. This work not only develops a high-performance catalyst for semi-hydrogenation of C_2_H_2_ but also provides an approach for the construction of effective catalytic structures based on unconventional SMSI.

## Methods

### Chemicals

Pd(acac)_2_ (>99.99%), OAM (90%), tri-n-octylphosphine (TOP, AR.), hexane (AR.), ethanol (AR.), ferric (III) acetylacetonate (Fe(acac)_3_, >99.99%), and *γ*-Al_2_O_3_ were obtained from Sigma Aldrich Corporate (USA) without further purification.

### Preparation of 4 nm Pd NPs

The Pd NPs were prepared by a modified method from previous work as following^27^: 70 mg of Pd(acac)_2_ was mixed with 15 mL of OAM in a 100 mL of four-neck flask under stirring. The mixture was then heated to 80 °C at a ramping rate of 5 °C min^−1^ and kept for 1 h under the protection of N_2_. A total of 0.5 mL of TOP was added to the solution. The mixture was further heated to 250 °C at a ramping rate of 5.6 °C min^−1^, and kept at this temperature for another 1 h before cooling down to room temperature. Subsequently, the mixture was transferred to a 50 mL of centrifuge tube, and 30 mL of ethanol was added. The Pd NPs were separated by centrifugation at 4656 × *g* for 10 min. Then, the Pd NPs were redispersed in 10 mL of hexane, and precipitated and washed by adding 30 mL of ethanol for two times. Finally, the Pd NPs were dispersed in 10 mL of hexane for further use.

### Preparation of Pd–FeO_x_ NPs

A total of 110 mg of Fe(acac)_3_ and 20 mL of OAM were added to a 100 mL four-neck flask. The mixture was heated to 90 °C at a ramping rate of 5 °C min^−1^ in N_2_. Subsequently, 12.5 mg of Pd NPs were added, followed by heating to 250 °C and kept there for 30 min. Afterward, the reaction temperature was raised to 300 °C and kept there for another 30 min before naturally cooling to room temperature. Then, 30 mL of ethanol was added to precipitate the Pd–FeO_x_ NPs, and then centrifuged at 4656 × *g* for 10 min. The Pd–FeO_x_ NPs was redispersed in 10 mL of hexane and washed by 30 mL of ethanol for two times. Finally, the Pd–FeO_x_ NPs were dispersed in 10 mL of hexane.

### Preparation of FeO_x_ NPs

The synthesis of FeO_x_ NPs is the same as the preparation of Pd–FeO_x_ NPs, without adding Pd NPs.

### Preparation of supported NPs

We employed a common-used insert material, *γ*-Al_2_O_3_, as the support to anchor the Pd–FeO_x_ NPs (or Fe_3_O_4_ NPs, or Pd NPs). Typically, 200 mg of *γ*-Al_2_O_3_ was dispersed in a mixture of 15 mL of hexane and 20 mL of ethanol under sonication. A total of 20 mg of prepared NPs in 5 mL of hexane was added into the solution dropwise under sonication. The final mixture was further sonicated for 2 h and then magnetically stirred overnight. Subsequently, the NPs/Al_2_O_3_ was separated by centrifugation at 6082 × *g* for 10 min, and washed by 20 mL of ethanol and hexane for two times. The final sample was dried at 50 °C under vacuum overnight.

### Preparation of Pd–Fe_3_O_4_–H and Pd–Fe_3_O_4_–A

The Pd–Fe_3_O_4_–H sample was prepared as follows: the Pd–FeO_x_ NPs/Al_2_O_3_ (ST2 NPs/Al_2_O_3_) was placed in a tube furnace and then heated to 300 °C at a ramping rate of 20 °C min^−1^ and kept there for 1 h under the atmosphere of 4% H_2_ in Ar atmosphere. The sample obtained was denoted as Pd–Fe_3_O_4_–H. The Pd amount was determined to be 0.171 wt.% by ICP. The Pd–Fe_3_O_4_–A sample was prepared by treating the Pd–FeO_x_ NPs/Al_2_O_3_ (ST2 NPs/Al_2_O_3_) in air under the same reaction condition.

### Preparation of Pd–Fe_3_O_4_–Re

The Pd–Fe_3_O_4_–Re sample was prepared by treating Pd–Fe_3_O_4_–H in the air for another 1 h.

### Preparation of Fe_3_O_4_–H

The Fe_3_O_4_–H sample was prepared by the same process of preparation of Pd–Fe_3_O_4_–H by using FeO_x_ NPs/Al_2_O_3_ instead of Pd–FeO_x_ NPs/Al_2_O_3_ .

### Preparation of control samples with different STs

Control samples with different ST were obtained by a similar synthesis process of Pd–FeO_x_ NPs using different amounts of Pd NPs seeds (37.5, 12.5, and 6.25 mg), the samples were respectively denoted as ST1 NPs, ST2 NPs, and ST3 NPs. The NPs were then deposited on the *γ*-Al_2_O_3_ and further treated in the atmosphere of 4% H_2_ in Ar for 1 h according to the same process of Pd–Fe_3_O_4_–H. (ST2 is the Pd–Fe_3_O_4_–H sample in this work).

### Preparation of control samples with different structures

The control samples with different structures were prepared according to a similar process of Pd–Fe_3_O_4_–H at different annealing temperatures. The annealing temperature was 200 °C, 300 °C, and 400 °C, and the corresponding samples were denoted as T200, T300, and T400 (T300 is the Pd–Fe_3_O_4_–H sample in this work).

### Characterization

The powder X-ray diffraction (XRD) patterns were collected on a PANalytical X’Pert Pro MPD diffractometer using an X’Celerator RTMS detector. HAADF-STEM and HR-STEM were performed on a Nion Ultra STEM 100 (operated at 100 kV). EELS spectra were collected on a high-resolution Gatan–Enfina ER with a probe size of 1.3 Å. TEM and high-angle annular bright-field scanning transmission electron microscopy (HAABF-STEM) were obtained on a Hitachi HD-200 with bright-field STEM detector operating at 200 kV.

The dispersion of the Pd was evaluated via pulse H_2_-Chemisorption with an Altamira Instruments (AMI-300) system. Before the measurements, ~100 mg catalyst was pretreated at 550 °C for 3 h under 50 sccm of Ar, followed by cooling down to desired temperature (i.e., −130 and 35 °C) under the same flow. Then pulses of 4% H_2_/Ar from a sample loop with a defined volume (~0.5 cc) were injected by switching a six-way valve until the eluted peak area of consecutive pulses was constant. The dispersion of Pd was calculated from the volume of H_2_.

INS experiments were performed at the VISION beamline of the Spallation Neutron Source, Oak Ridge National Laboratory. The Pd–Fe_3_O_4_–H sample was first treated under vacuum at 600 °C for 12 h. It was then loaded in an aluminum sample holder in a helium glovebox. The sample holder was attached to a gas-loading sample stick connected to a gas panel. The blank sample was first measured at −268 °C for 3 h to collect baseline spectrum. H_2_ gas was then introduced in situ at −238 °C, followed by heating of the sample to −98 °C for reaction. The system was then cooled back to −268 °C to measure the reacted spectrum. The difference spectrum (reacted minus baseline) shows the signal associated with the hydride species formed during the reaction. The CO DRIFTS results were obtained on a Nicolet 670 Fourier Transform Infrared Spectrometer with an MCT detector by the following process: each sample (~15 mg) was loaded and then pretreated at 200 °C under Ar for 30 min. Afterward, the sample was cooled down to −120 °C to conduct CO adsorption. When the temperature reached −120 °C, the background was measured and then CO adsorption was conducted for 30 min as followed by desorption with Ar for 10 min (CO desorbed within 1 min after flow Ar). XPS characterization was performed on a PHI VersaProbe III scanning XPS microscope using a monochromatic Al K-alpha X-ray source (1486.6 eV). XPS spectra were acquired with 200 µm/50 W/15 kV X-ray settings and dual-beam charge neutralization. All binding energies were referenced to Al 2*p* peak at 74.8 eV.

### Catalytic performance tests

The hydrogenation of C_2_H_2_ was carried out in a tubular quartz reactor with a 0.25-inch diameter. In a typical run, ~15 mg of catalyst was mixed with 150 mg of 60–80 mesh quartz sand and placed in the center of the reactor. The catalyst bed was held by quartz wool at both ends and the reactor was loaded in a vertical furnace (Carbolite Gero). The catalyst was purged with He for 30 min at a flow rate of 20 sccm prior to the reaction under room temperature. Then, the reactor was heated to the desired temperature (i.e., 30–80 °C), followed by feeding the gas mixture (i.e., 0.6 sccm C_2_H_2_, 3 sccm H_2_ balanced with He) at a total flow rate of 50 sccm. The exit gas mixture was analyzed on-line by a ThermoStar Mass Spectrometry (Pfeiffer).

The conversion and selectivity were calculated by using Eqs. (1) and (2):

1 $$ {{{\rm{C}}_2{\rm{H}}_2}}\;{{\rm{Conversion}}}\left( {\mathrm{\% }} \right) = \left( {1 - \frac{{{{{\rm{X}}_{{\rm{C}}_2{\rm{H}}_2,{\rm{out}}}}}}}{{{{{\rm{X}}_{{\rm{C}}_2{\rm{H}}_2,{\rm{in}}}}}}}} \right) \times 100{\mathrm{\% }}$$

2 $${\mathrm{Selectivity}}\left( {\mathrm{\% }} \right) = \frac{{{{{\rm{X}}_{{\rm{C}}_2{\rm{H}}_4,{\rm{out}}}}}}}{{{{{\rm{X}}_{{\rm{C}}_2{\rm{H}}_2,{\rm{in}}}}} - {{{\rm{X}}_{{\rm{C}}_2{\rm{H}}_2,{\rm{out}}}}}}} \times 100{\mathrm{\% }}$$

whereas in/out refers to the concentration measured in the inlet/outlet port.

Reaction orders with respect to H_2_ and C_2_H_2_ were calculated by the differential method. The corresponding conversion is maintained below 20% to ensure a true kinetic regime. Apparent activation energy is calculated by the Arrhenius equation.

### DFT calculation

The density functional theory calculations were performed with the Vienna Ab Initio Simulation Package (VASP)^49,50^. The on-site Coulomb interaction was included with the DFT + U method by Dudarev et al.^51^ in VASP using a Hubbard parameter *U* = 3.8 eV for the Fe atom. The Perdew–Burke–Ernzerhof^52^ functional form of generalized-gradient approximation was used for electron exchange and correlation energies. The projector augmented-wave method was used to describe the electron–core interaction^49,53^. A kinetic energy cutoff of 450 eV was used for the plane waves. A 3 × 2 × 1 sampling of Brillouin zone using a Monkhorst-Pack scheme was used^54^. A vacuum layer of 15 Å was added for the surface slabs along the *z*-direction; the slab contains a total of four layers, with the bottom two layers fixed in their bulk positions.

### XAFS data collection and processing

Approximately 20 mg of sample was enclosed in a nylon washer of 4.953 nm inner diameter and sealed on one side with transparent “Scotch” tape. The sample was pressed by hand to form a uniform pallet, then sealed on the open side with a tape. XAFS investigation were performed at beamline 10ID-B of the Advanced Photon Source at Argonne National laboratory^55^. Spectra were collected at the iron K-edge (7112 eV) and palladium K-edge (24,350 eV) in transmission mode, with an iron and palladium foil as a reference for energy calibration, respectively. All spectra were collected at room temperature and ten scans were collected for each sample. All data were processed and analyzed using the Athena and Artemis program of the IFFEFFIT package^56^ based on FEFF 6.0. Reference foil data were aligned to the first zero-crossing of the second derivative of the normalized *μ*(E) data, which was subsequently calibrated to the literature E_0_ for each Fe K-edge and Pd K-edge. The background was removed, and the data were assigned a Rbkg value of 1.0 prior to normalizing to obtain a unit edge step. All data were initially fit with k-weighting of 1, 2, and 3 then finalized with k^3^-weighting in R-space. A fit of the Pd foil and Fe foil was used to determine S_0_^2^ for each sample. Structure models used to fit the data sets were obtained from crystal structure of iron oxide and DFT calculation. Structure parameters that were determined by the fits include the degeneracy of the scattering path (N_degen_), the change in Reff, the mean square relative displacement of the scattering element(σ^2^_i_), and the energy shift of the photoelectron(ΔE_0_). k^3^-weighting in R-space. Initial fitting was conducted using crystal structure from crystal database. The simulated models were obtained from DFT calculation and scattering paths of selected scattered atom (Fe, Pd) were generated through FEFF calculation. The WT method was adapted for a quantitative analysis of the backscattering atom in the higher coordination shells with EvAX code^57^.

## Supplementary information

Supplementary Information

Peer Review File

## Data Availability

The data that support the plots within this paper and other findings of this study are available from the corresponding author upon reasonable request. Source data are provided with this paper.

## Source data

Source Data
