# Supplementary material for: Harnessing strong metal–support interactions via a reverse route
Source: Nat Commun. 2020 Jun 16;11:3042. doi: 10.1038/s41467-020-16674-y (PMC7297808; doi:10.1038/s41467-020-16674-y)
Supplement: Supplementary file 1 — Supplementary Information [file 41467_2020_16674_MOESM1_ESM.pdf]

Supplementary information

Harnessing strong metal-support interactions via a reverse route

Wu et al.

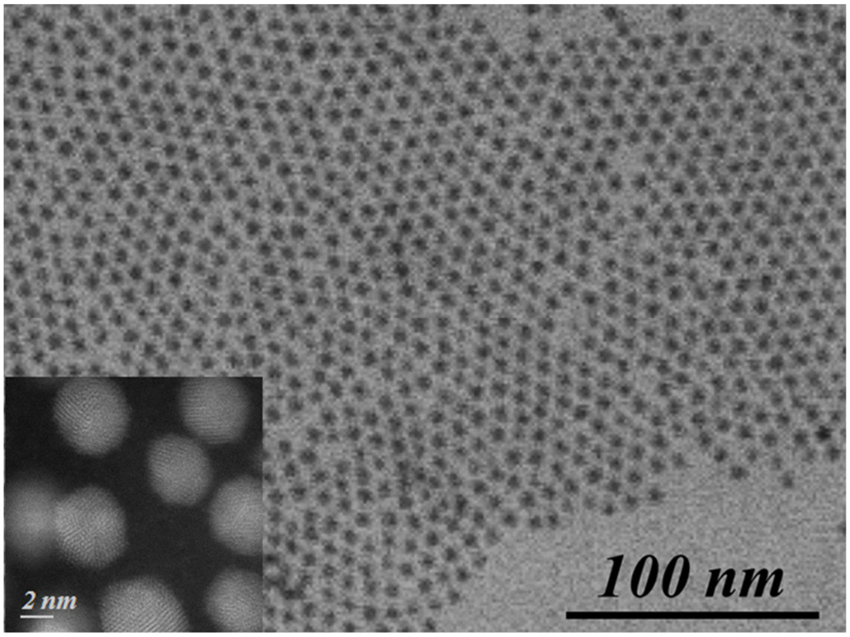

Supplementary Figure 1. TEM and HAADF-STEM (inset) images of the prepared Pd NPs.

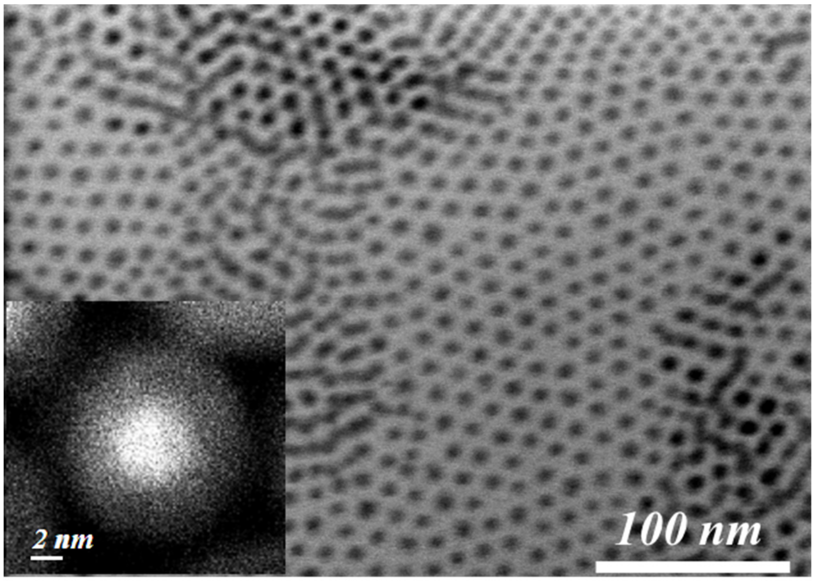

Supplementary Figure 2. TEM and HAADF-STEM (inset) images of the Pd-FeO_x NPs.

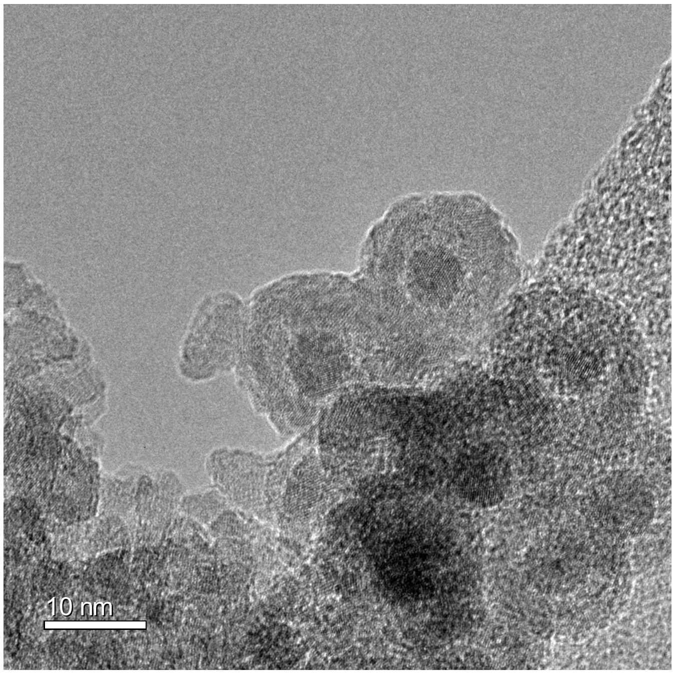

Supplementary Figure 3. The HAABF-STEM image of the Pd-FeO$_x$ NPs on γ-Al_2O_3.

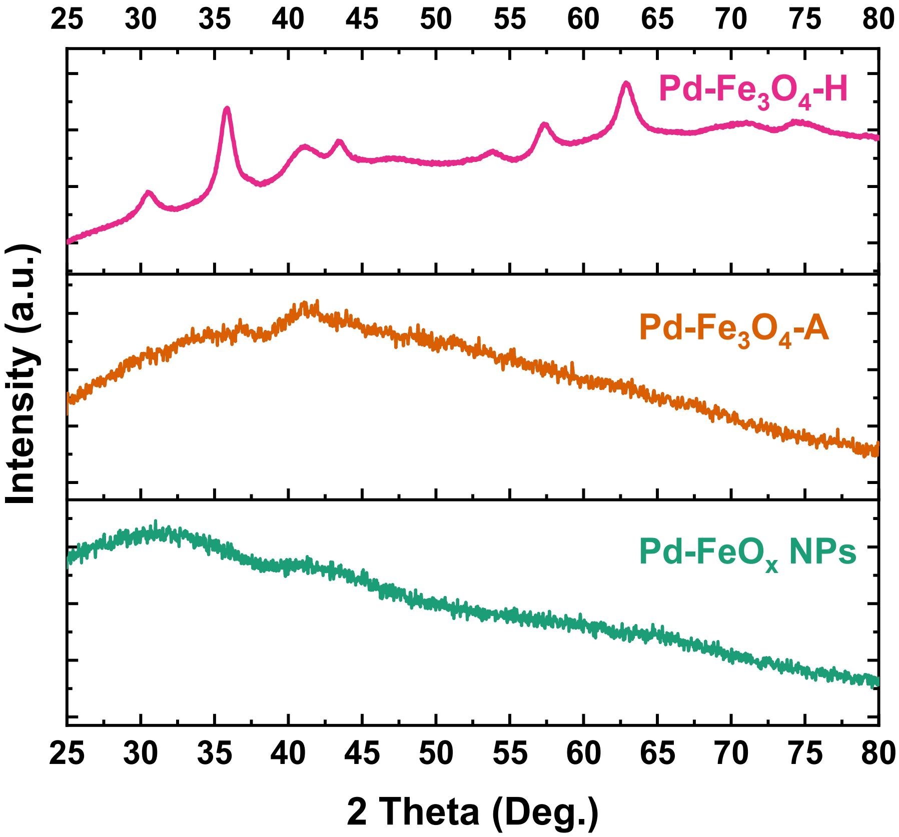

Supplementary Figure 4. XRD patterns of Pd-FeO$_x$ NPs, Pd-Fe_3O_4-A, and Pd-Fe_3O_4-H.
Source data are provided as a Source Data file.

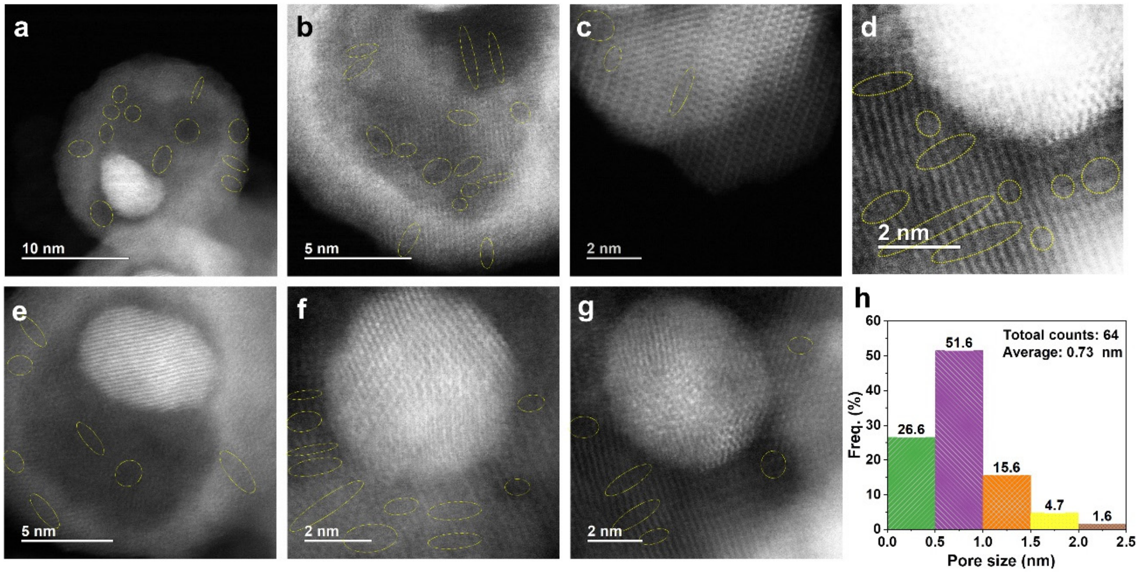

Supplementary Figure 5. a-g) HR-STEM images of Pd-Fe_3O_4-H and h) the pore size distribution.

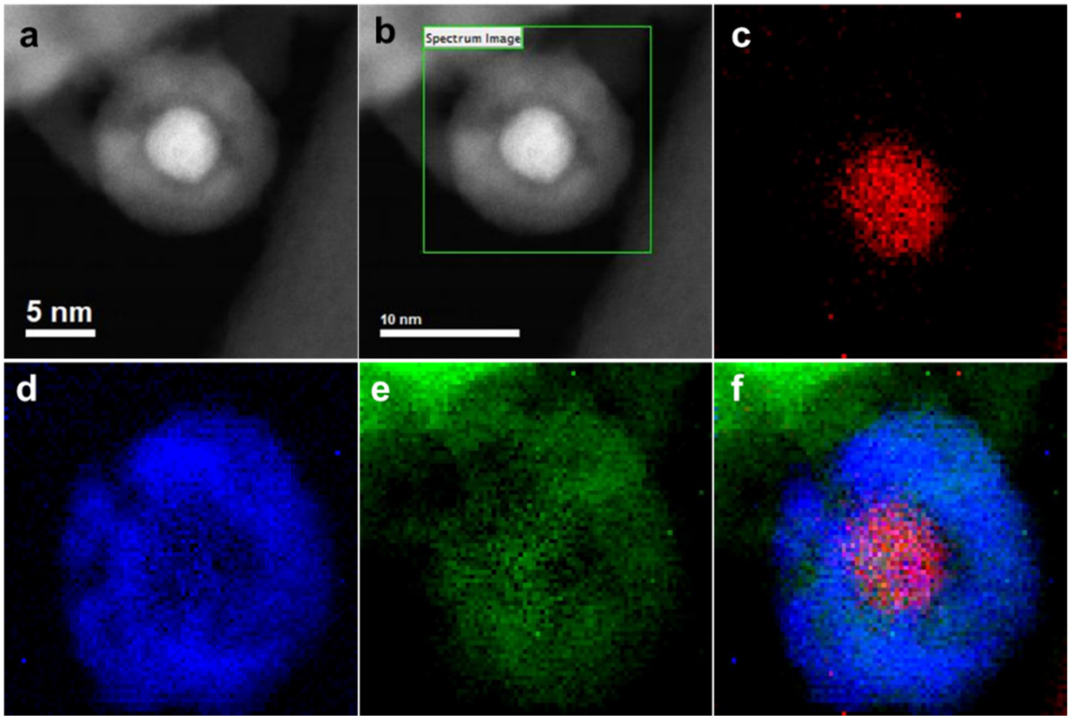

Supplementary Figure 6. STEM characterization of the Pd-Fe_3O_4-A sample. a) an STEM image of Pd-Fe_3O_4-A; c-f) corresponding EELS mapping characterization of the selected section in Supplementary Figure 6b; c) Pd; d) Fe; e) O; and f) overlapped figure. The scale bars in Supplementary Figures 6c-f are the same as that in Supplementary Figure 6b.

Supplementary Table 1. Curve-fitting results of Pd K-edge EXAFS spectra using theoretical crystal structure (R = effective bond distance, σ^2 = mean-squared relative displacement, parentheses = error)

		R / Å	Coordination Number	σ^2	E_0	R factor
$Pd\text{-}Fe_3O_4\text{-}H$	Pd-O	1.903 (0.046)	2 (0.004)	0.0057(0.0020)	4.819 (0.697)	0.020
	Pd-Fe	2.701 (0.067)	3 (0.004)	0.0120(0.0030)		
	Pd-Pd	2.791 (0.027)	6 (0.003)	0.0064(0.0056)		
$Pd\text{-}Fe_3O_4\text{-}A$	Pd-O	1.857 (0.097)	3 (0.028)	0.0073(0.0010)	5.13 (0.420)	0.029
	Pd-Fe	2.603 (0.067)	3 (0.028)	0.0025(0.0027)		
	Pd-Pd	2.801 (0.067)	4 (0.92)	0.0037(0.0010)		

Supplementary Table 2. Curve-fitting results of Fe K-edge EXAFS spectra using theoretical crystal structure

		R / Å	Coordination Number	σ^2	E_0	R factor
$Pd\text{-}Fe_3O_4\text{-}H$	Fe=O	-	-	-	4.231(0.124)	0.012
	Fe-O	2.09 (0.016)	3 (1.4)	0.008 (0.016)		
	Fe-Fe	3.06 (0.012)	4 (0.005)	0.007 (0.005)		
$Pd\text{-}Fe_3O_4\text{-}A$	Fe=O	-	-	-	4,523(0.452)	0.021
	Fe-O	2.09 (0.016)	3.2 (1.012)	0.008 (0.005)		
	Fe-Fe	3.04 (0.009)	2 (0.012)	0.007 (0.021)		

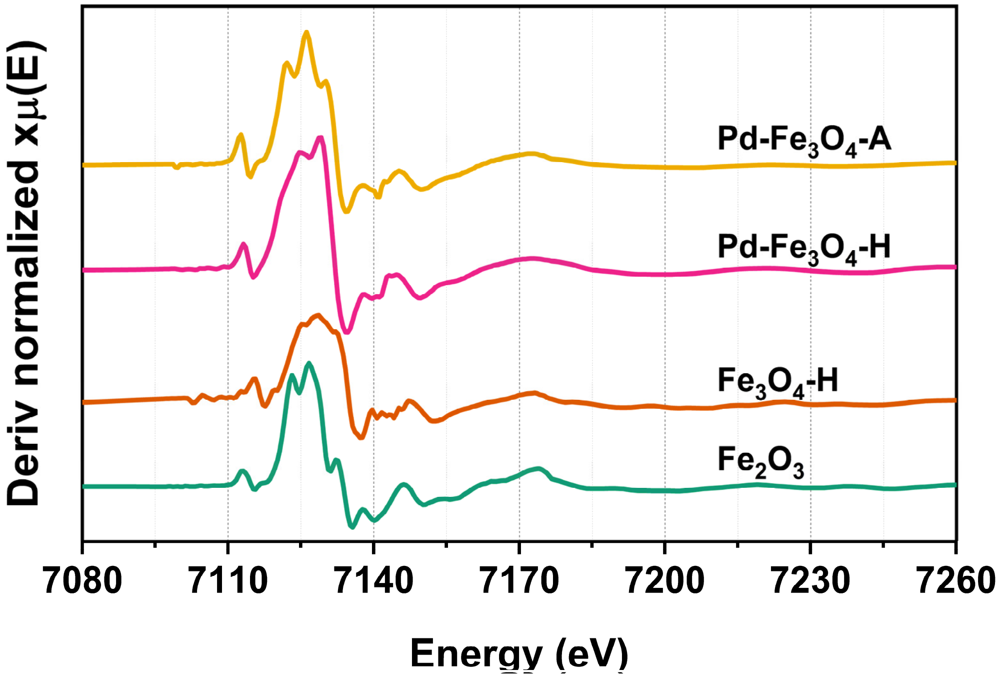

Supplementary Figure 7. Fe K-edge 1st derivative XANES of $Pd\text{-}Fe_3O_4\text{-}A$, $Pd\text{-}Fe_3O_4\text{-}H$, Fe_3O_4, and Fe_2O_3. Source data are provided as a Source Data file.

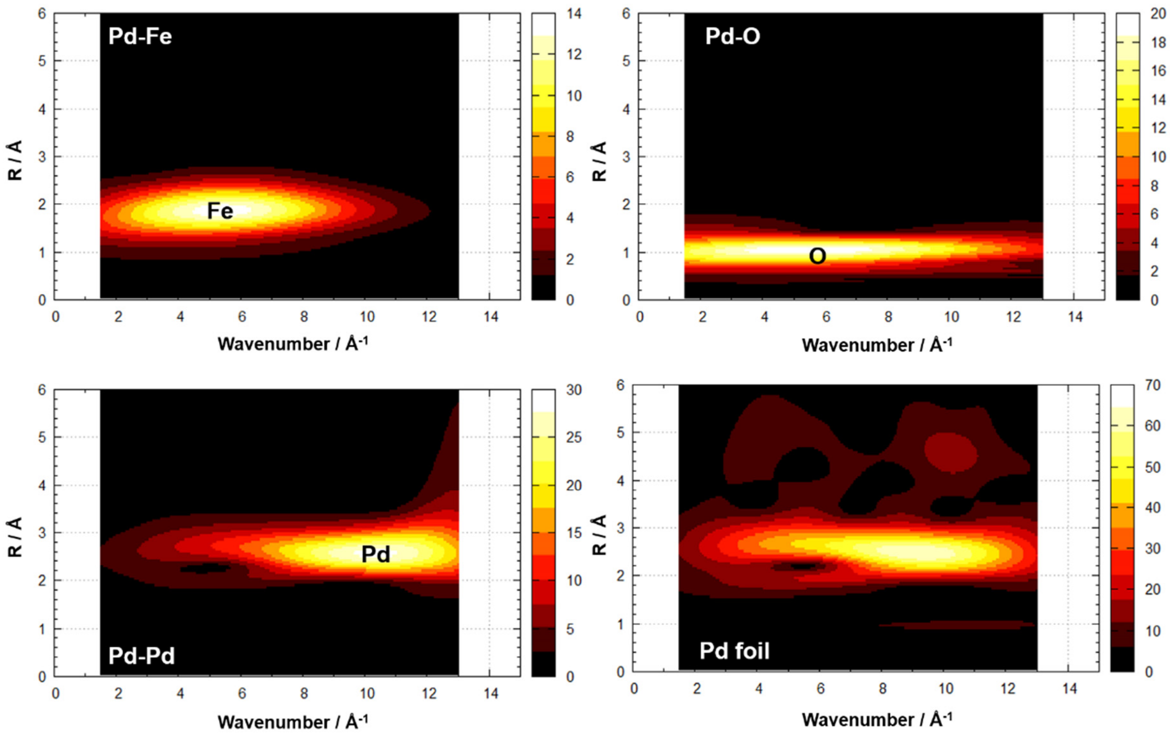

Supplementary Figure 8. Standard WT EXAFS images of Pd-Fe, Pd-O, Pd-Pd, and Pd foil.

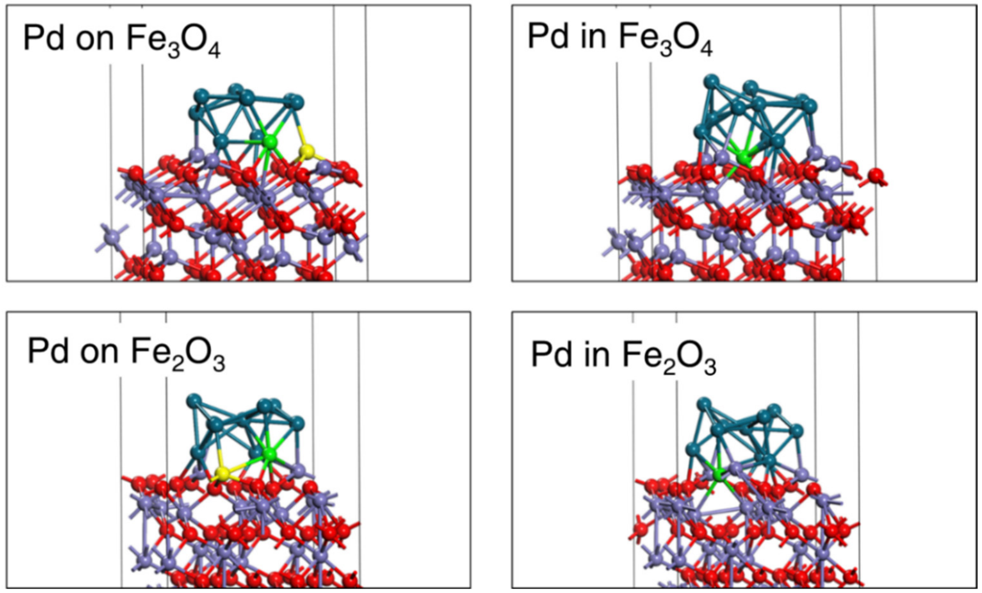

Supplementary Figure 9. DFT optimized structures of palladium atoms (blue) with different iron oxide (purple-iron, red-oxygen). Two different scenarios for palladium were applied on Fe_3O_4 and Fe_2O_3. All palladium atoms are on the Fe_3O_4 or Fe_2O_3 (Left top and bottom, respectively) and a palladium atom (green color) is in the Fe_3O_4 or Fe_2O_3 substituted oxygen atom from the iron oxide (Right top and bottom, respectively). Highlighted atoms (green-Pd, yellow-Fe) are used as a core atom for Fe K-edge and Pd K-edge, respectively to generate the scattering path.

Supplementary Table 3. FEFF calculated scattering path generated by using simulated structure model (Pd on Fe_2O_3)

No.	Pd K-edge		Fe K-edge	
	Scattering Path	R_{eff}	Scattering Path	R_{eff}
1	Pd-O1	2.093	Fe-O1	1.898
2	Pd-O2	2.169	Fe-O2	1.933
3	Pd-Pd1	2.529	Fe-O3	2.010
4	Pd-Pd2	2.603	Fe-O-O	3.479
5	Pd-Fe	2.705	Fe-Fe	3.492

Supplementary Table 4. FEFF calculated scattering path generated by using simulated structure model (Pd on Fe_3O_4)

No.	Pd K-edge		Fe K-edge	
	Scattering Path	R_{eff}	Scattering Path	R_{eff}
1	Pd-O1	2.039	Pd-O1	1.967
2	Pd-Pd	2.528	Pd-O2	2.012
3	Pd-Fe1	2.862	Pd-O3	2.099
4	Pd-Fe2	2.997	Fe-O4	2.128
5	Pd-O2	3.063	Fe-Fe1	2.919

Supplementary Table 5. FEFF calculated scattering path in Pd K-edge generated by using simulated structure model

No.	Pd in Fe_2O_3		Pd in Fe_3O_4	
	Scattering Path	R_{eff}	Scattering Path	R_{eff}
1	Pd-O1	2.542	Pd-Fe1	2.500
2	Pd-Pd1	2.623	Pd-Fe2	2.582
3	Pd-Fe	2.671	Pd-Fe3	2.683
4	Pd-Pd2	2.665	Pd-Pd1	2.750
5	Pd-Pd3	2.764	Pd-O	2.950

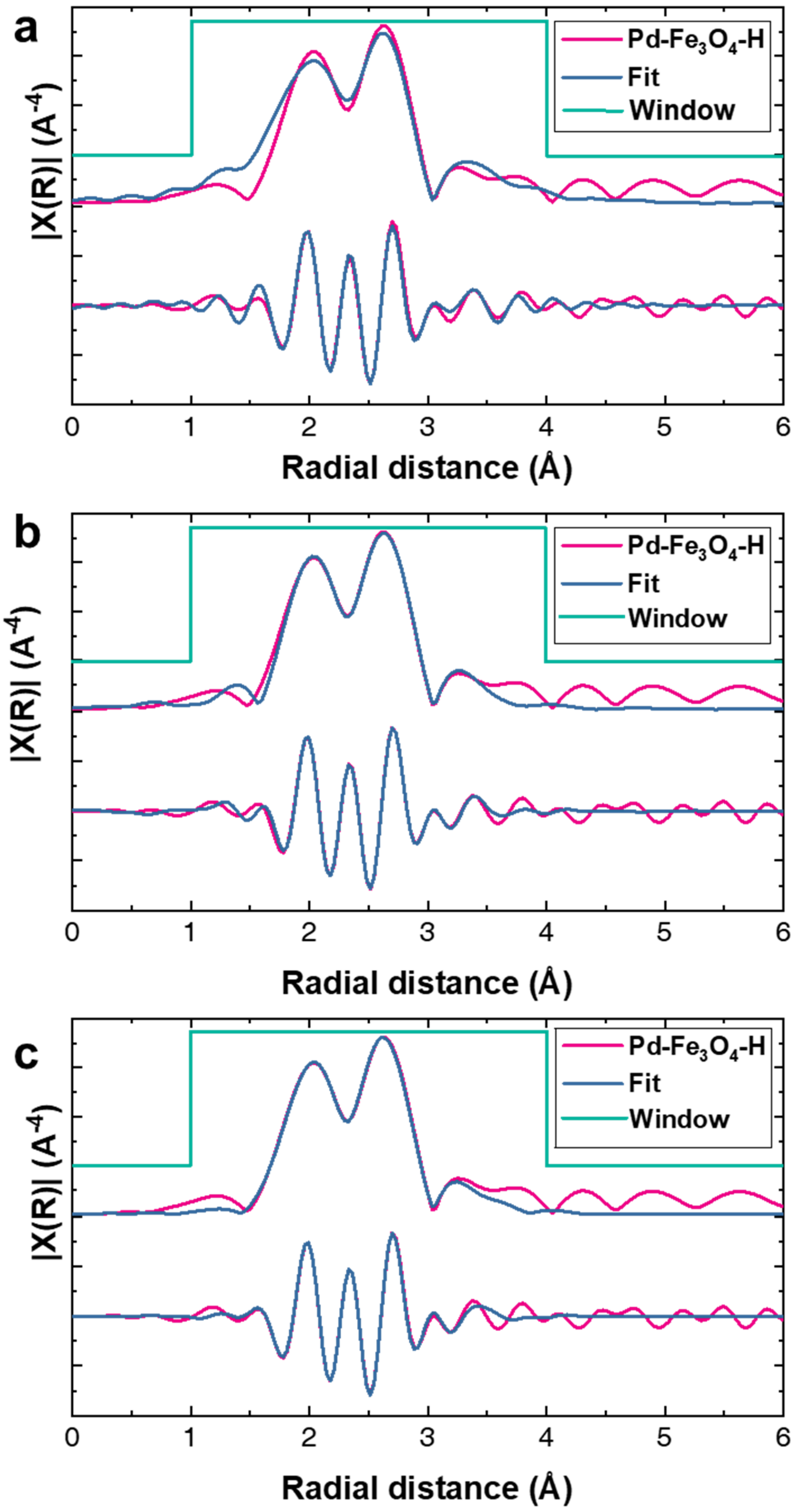

Supplementary Figure 10. Pd K-edge EXAFS fitting of Pd-Fe_3O_4-H. a) Pd on Fe_2O_3 surface; b) Pd on Fe_3O_4 surface; and c) Pd in Fe_3O_4 surface. Source data are provided as a Source Data file.

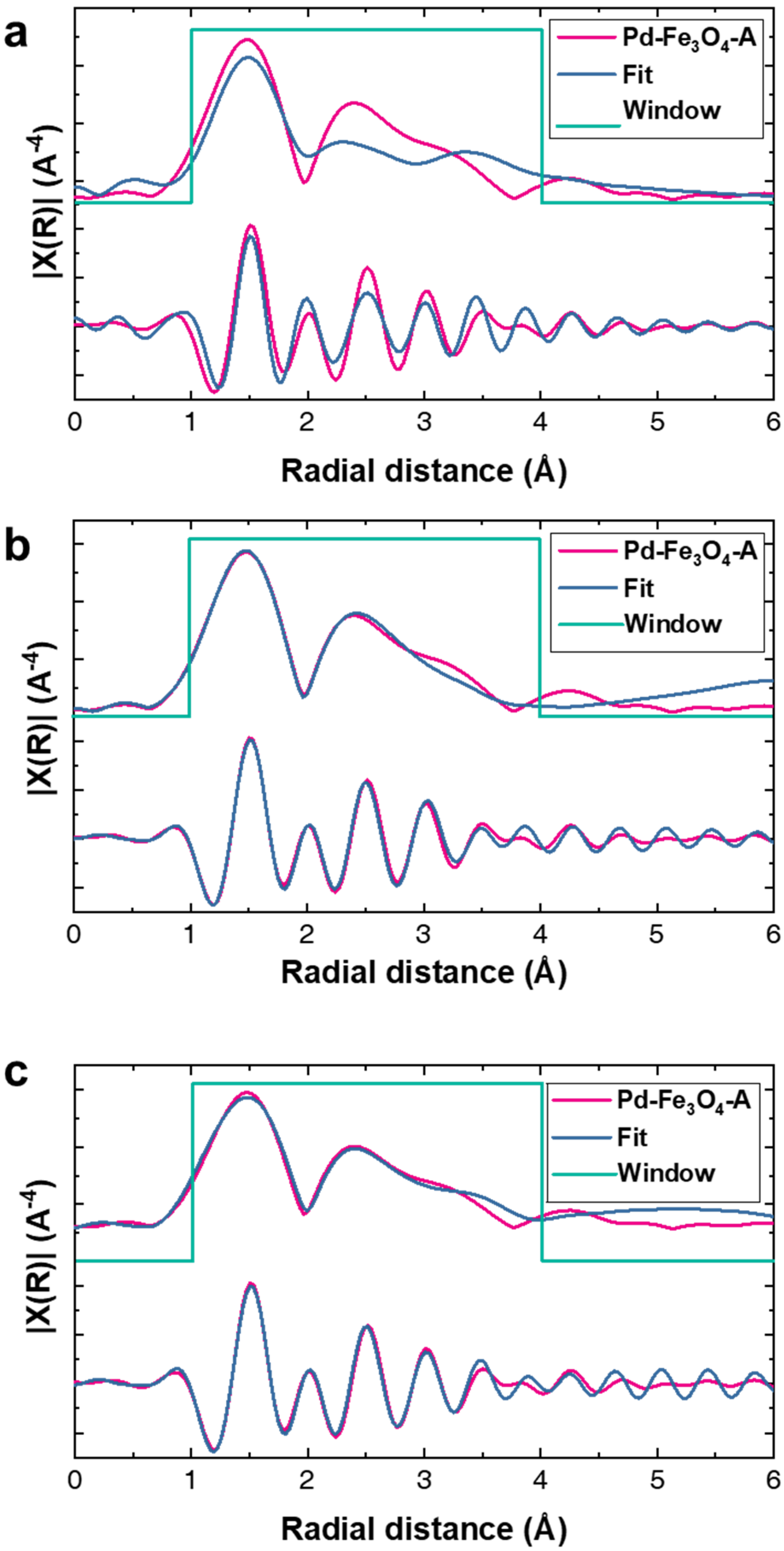

Supplementary Figure 11. Pd K-edge EXAFS fitting of Pd-Fe_3O_4-A. a) Pd in Fe_2O_3 surface; b) Pd on Fe_2O_3 surface; and c) Pd on Fe_3O_4 surface. Source data are provided as a Source Data file.

Supplementary Table 6. Curve-fitting results of Pd K-edge EXAFS spectra for Pd-Fe_3O_4-H using simulated DFT structure model (ΔR = effective bond distance difference, σ^2 = mean-squared relative displacement, parentheses = error)

Simulated Model	Pd on Fe_2O_3	Pd on Fe_3O_4	Pd in Fe_3O_4
E_0	3.9 (0.22)	4.01 (0.33)	4.13 (0.24)
S_0^2	1.24	1.98	1.54
R-factor	0.042	0.019	0.025
ΔR_1	-0.001(0.005)	0.003(0.003)	0.006(0.003)
σ_1^2	0.002(0.0002)	0.001(0.0005)	0.004(0.005)
ΔR_2	0.003(0.012)	-0.004(0.002)	-0.003(0.005)
σ_2^2	0.010(0.018)	0.009(0.002)	0.006(0.014)
ΔR_3	-0.002(0.005)	-0.001(0.042)	0.005(0.081)
σ_3^2	0.082(0.007)	0.003(0.008)	0.001(0.002)
ΔR_4	0.002(0.003)	0.042(0.087)	0.003(0.002)
σ_4^2	0.006(0.002)	0.003(0.001)	0.009(0.005)
ΔR_5	-0.02(0.008)	0.012(0.005)	-0.001(0.008)
σ_5^2	0.003(0.009)	0.001(0.008)	0.012(0.001)

Supplementary Table 7. Curve-fitting results of Pd K-edge EXAFS spectra for Pd-Fe_3O_4-A using simulated DFT structure model (ΔR = effective bond distance difference, σ^2 = mean-squared relative displacement, parentheses = error)

Simulated Model	Pd on Fe_2O_3	Pd on Fe_3O_4	Pd in Fe_3O_4
E_0	5.92 (0.18)	4.73 (0.76)	9.35 (0.31)
S_0^2	1.32	1.09	0.79
R-factor	0.029	0.015	0.031
ΔR_1	0.001(0.002)	0.003(0.001)	0.033(0.042)
σ_1^2	0.007(0.013)	0.003(0.0001)	0.016(0.002)
ΔR_2	-0.042(0.013)	-0.002(0.001)	-0.015(0.035)
σ_2^2	0.001(0.039)	0.002(0.001)	0.097(0.018)
ΔR_3	0.007(0.001)	-0.001(0.042)	0.029(0.062)
σ_3^2	0.006(0.002)	0.003(0.003)	0.002(0.008)
ΔR_4	0.006(0.001)	0.001(0.007)	0.007(0.001)
σ_4^2	0.003(0.001)	0.007(0.002)	0.003(0.001)
ΔR_5	-0.020(0.005)	0.007(0.007)	0.02(0.008)
σ_5^2	0.002(0.001)	0.003(0.00`)	0.012(0.001)

* Mixed model used first 4 scattering paths from Pd on Fe_2O_3 and Pd on Fe_3O_4

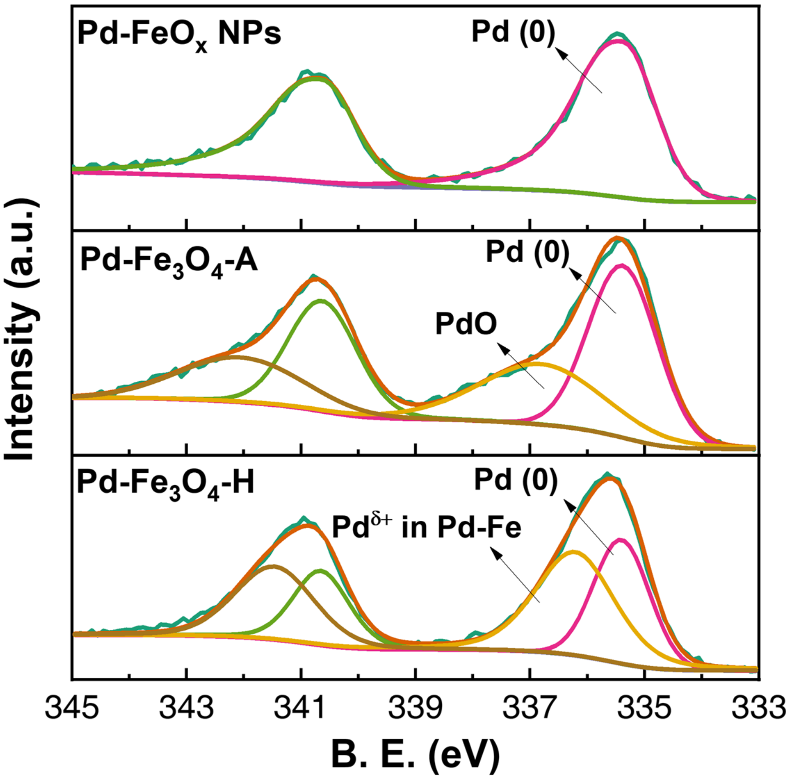

Supplementary Figure 12 High-resolution Pd *3d* XPS spectra of Pd-FeO$_x$ NPs, Pd-Fe$_3$O$_4$-H and Pd-Fe$_3$O$_4$-A. Source data are provided as a Source Data file.

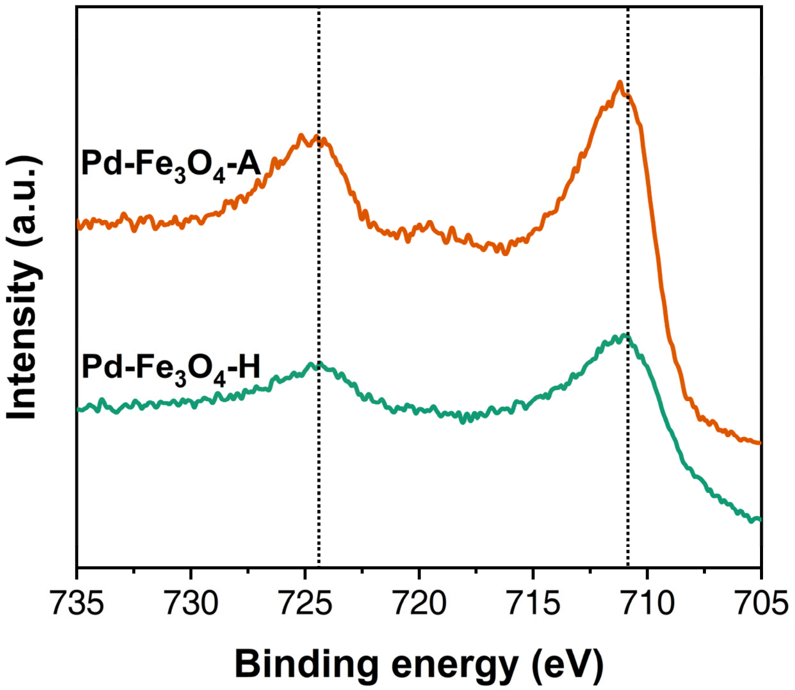

Supplementary Figure 13 High-resolution Fe *2p* XPS spectra of Pd-Fe$_3$O$_4$-H and Pd-Fe$_3$O$_4$-A. Source data are provided as a Source Data file.

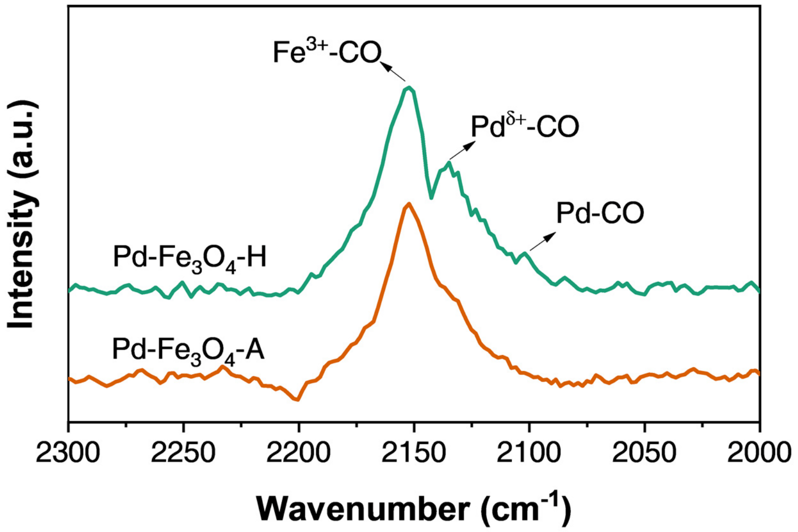

Supplementary Figure 14 CO DRIFTS of Pd-Fe_3O_4-H and Pd-Fe_3O_4-A. Source data are provided as a Source Data file.

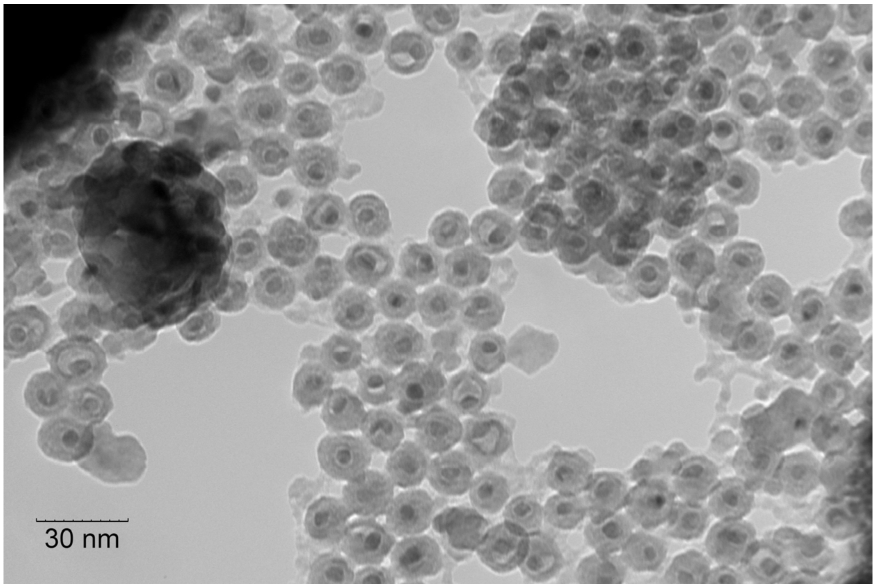

Supplementary Figure 15 The TEM image of Pd-Fe_3O_4-Re.

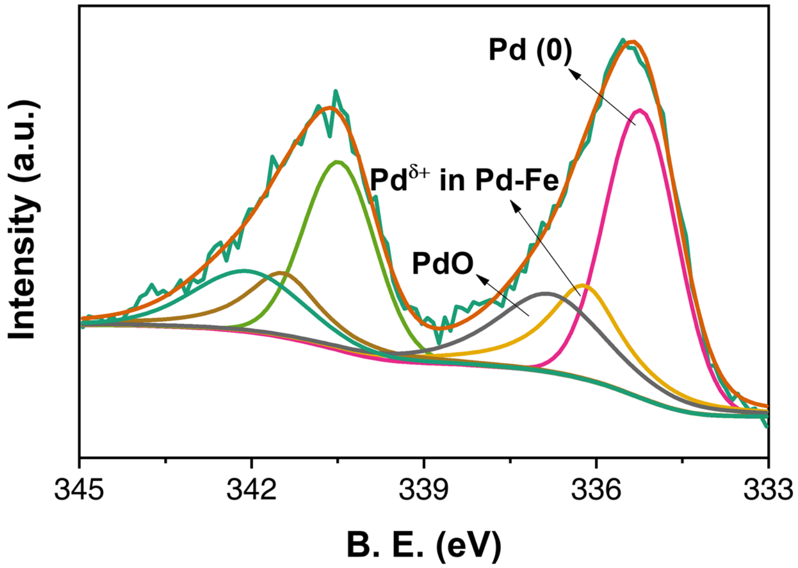

Supplementary Figure 16 High-resolution Pd *3d* XPS spectrum of Pd-Fe_3O_4-Re. Source data are provided as a Source Data file.

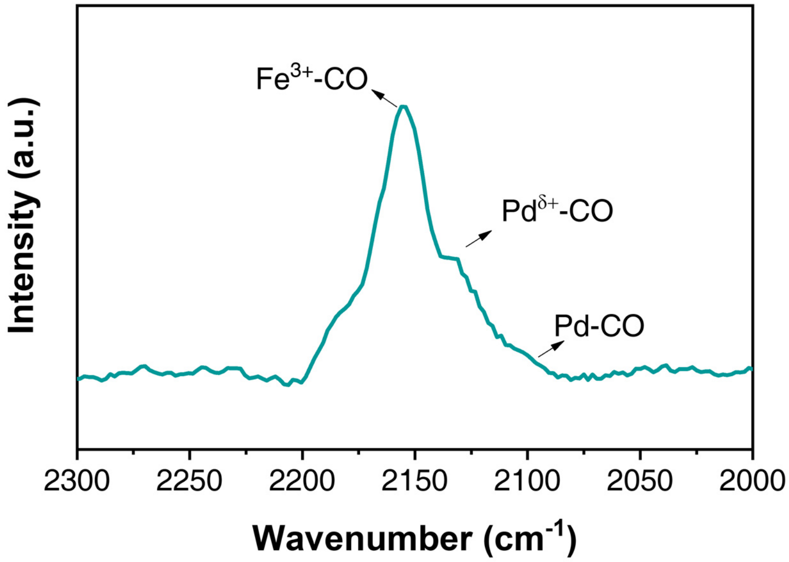

Supplementary Figure 17 CO DRIFTS of Pd-Fe_3O_4-Re. Source data are provided as a Source Data file.

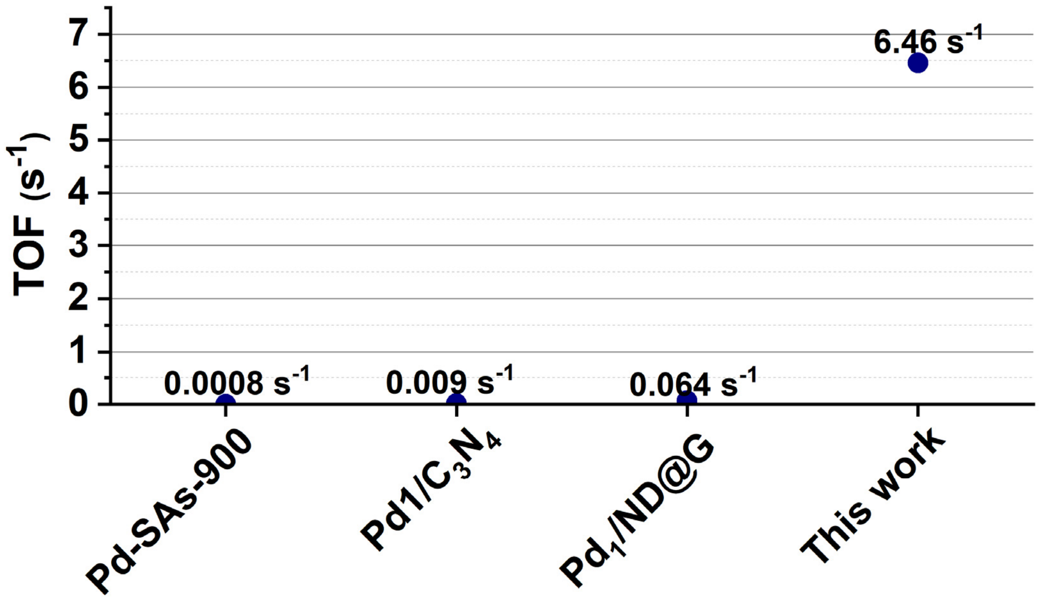

Supplementary Figure 18. A comparison of TOFs in acetylene hydrogenation over Pd-based catalysts at the reaction temperature of 80 °C between previous works and this work.
Sample in Ref. 1 is the Pd single atoms on carbon support (Pd-SAs-900), which is obtained by thermally treating Pd-NPs@ZIF-8 sample[1]; sample in Ref. 2 is carbon nitride supported Pd single atom catalyst (Pd1/C_3N_4)[2]; sample in Ref. 3 is the nanodiamond/graphene hybrid supported Pd single atom catalyst (Pd_1/ND@G)[3].

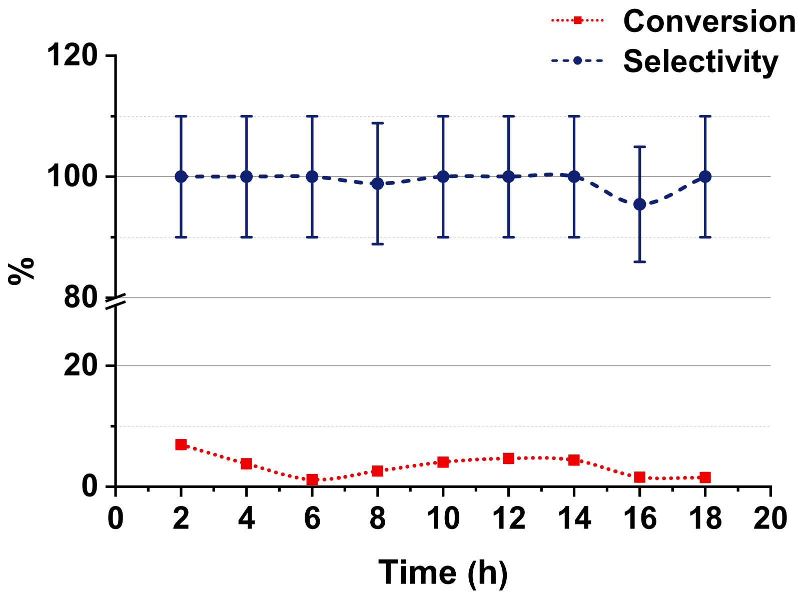

Supplementary Figure 19. Stability investigation of the Pd-Fe_3O_4-H catalyst at a low conversion level.
Reaction conditions: *m* (catalyst) = 30 mg; *v* (gas) = 50 sccm (C_2H_2 is 0.6 sccm, H_2 is 3 sccm, balanced with He); *T* = 40 °C. Error bars represent the instrumental error (± 10 %). Source data are provided as a Source Data file.

Supplementary Table 8. Dispersion and Pd particle sizes of Pd-Fe_3O_4-H and Pd/Al_2O_3 determined by H_2-chemisorption at different temperatures.

	Pd-Fe_3O_4-H		Pd	
Temp (°C)	35	- 130	35	- 130
Dispersion (%)	24.4	26.7	72	7.6
Particle size (nm)	4.6	4.2	1.6	14.8
Particle size from STEM (nm)	5.5		-	

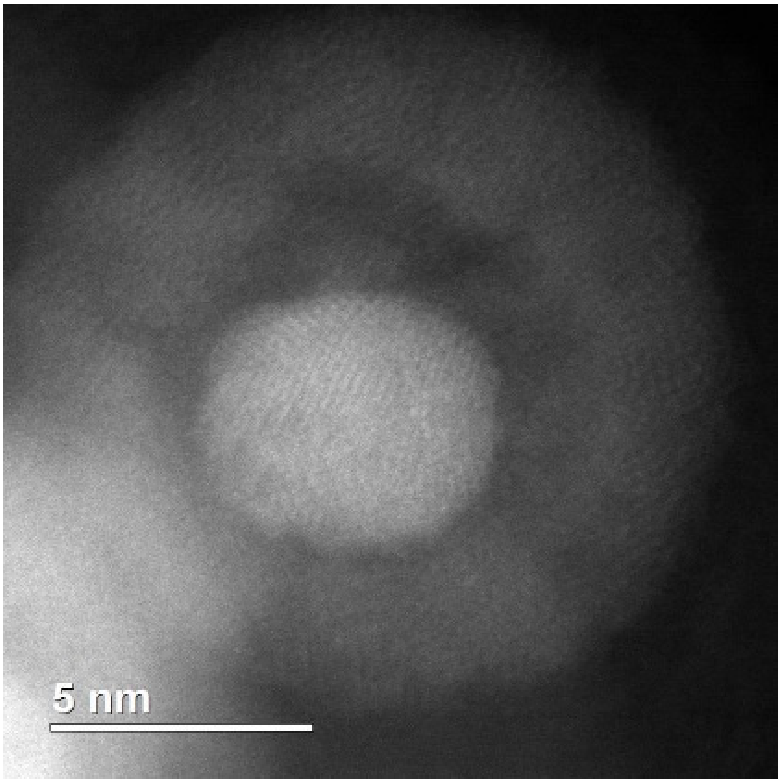

Supplementary Figure 20. The HAADF-STEM image of the prepared T200 sample.

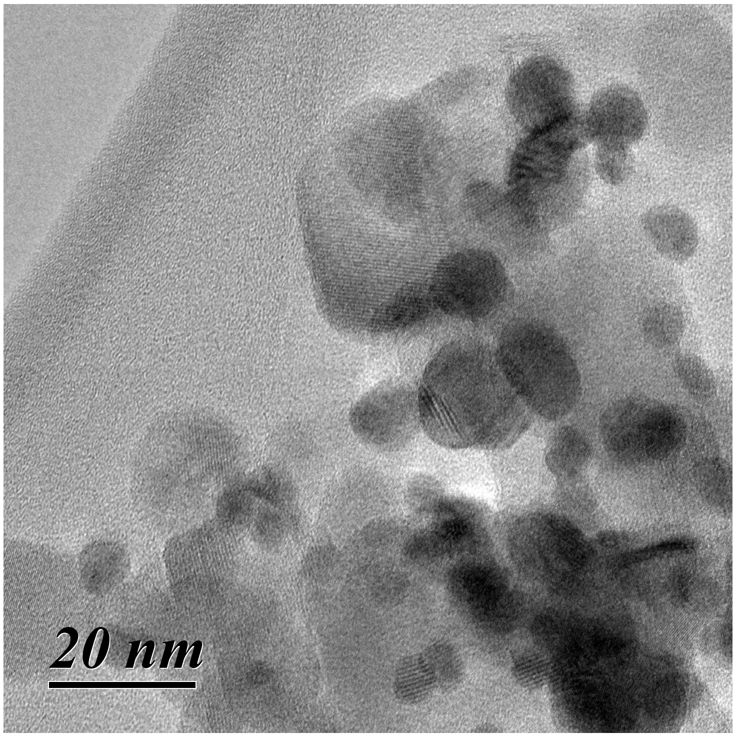

Supplementary Figure 21. The TEM image of T400 sample.

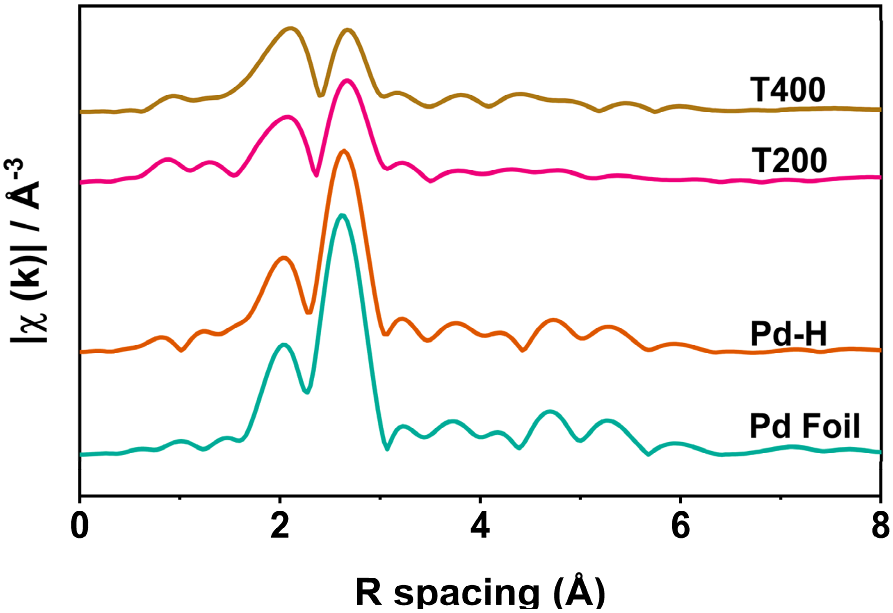

Supplementary Figure 22. Pd K-edge EXAFS of T200, T400 and references. Source data are provided as a Source Data file.

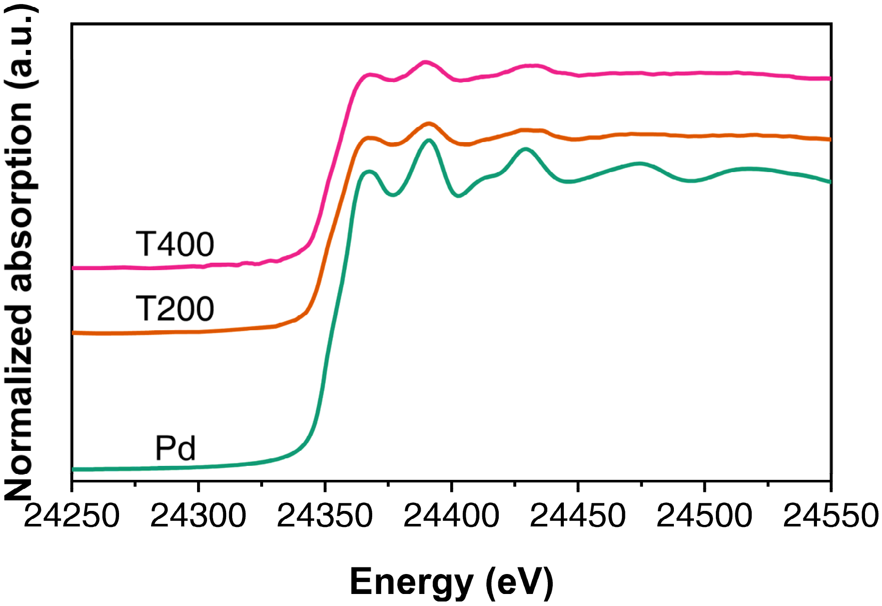

Supplementary Figure 23. Pd K-edge XANES of T200, T400 and Pd reference. Source data are provided as a Source Data file.

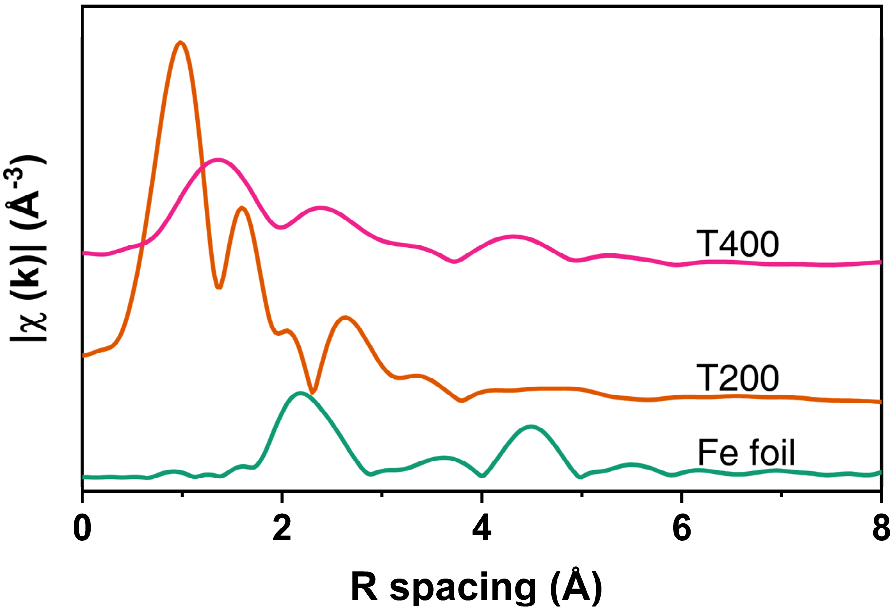

Supplementary Figure 24. Fe K-edge EXAFS of T200, T400 and Fe reference. Source data are provided as a Source Data file.

Supplementary Table 9. Curve-fitting results of Pd K-edge EXAFS spectra using theoretical crystal structure (R = effective bond distance, σ^2 = mean-squared relative displacement, parentheses = error)

		R / Å	Coordination Number	σ^2	E_0	R factor
T200	Pd-O	1.904 (0.062)	0.5 (0.153)	0.0039(0.0019)	6.707 (0.920)	0.023
	Pd-Fe	2.716 (0.012)	2 (0.005)	0.0130(0.0050)		
	Pd-Pd	2.795 (0.004)	6 (0.31)	0.0041(0.0019)		
T400	Pd-O	1.923 (0.095)	2 (0.538)	0.0028(0.0013)	5.41 (0.336)	0.027
	Pd-Fe	2.703 (0.073)	2 (0.006)	0.0057(0.0030)		
	Pd-Pd	2.791 (0.056)	4 (0.939)	0.0061(0.0022)		

Supplementary Table 10. Curve-fitting results of Fe K-edge EXAFS spectra using theoretical crystal structure

		R / Å	Coordination Number	σ^2	E_0	R factor
T200	Fe=O	1.863 (0.025)	4 (0.003)	0.012 (0.09)	3.013(0.135)	0.023
	Fe-O	2.103 (0.011)	4 (0.003)	0.005 (0.0035)		
	Fe-Fe	2.986 (0.015)	4 (0.009)	0.0093 (0.007)		
T400	Fe=O	-	-	-	3.241(0.243)	0.027
	Fe-O	1.91 (0.011)	2 (0.11)	0.03 (0.002)		
	Fe-Fe	3.10 (0.016)	2 (0.26)	0.001 (0.008)		

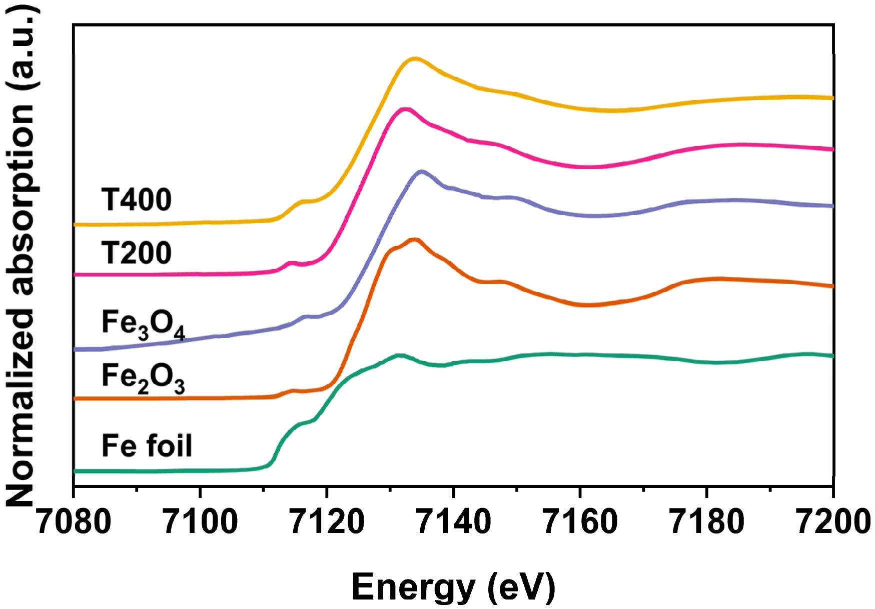

Supplementary Figure 25. Fe K-edge XANES of T200, T400 and references. Source data are provided as a Source Data file.

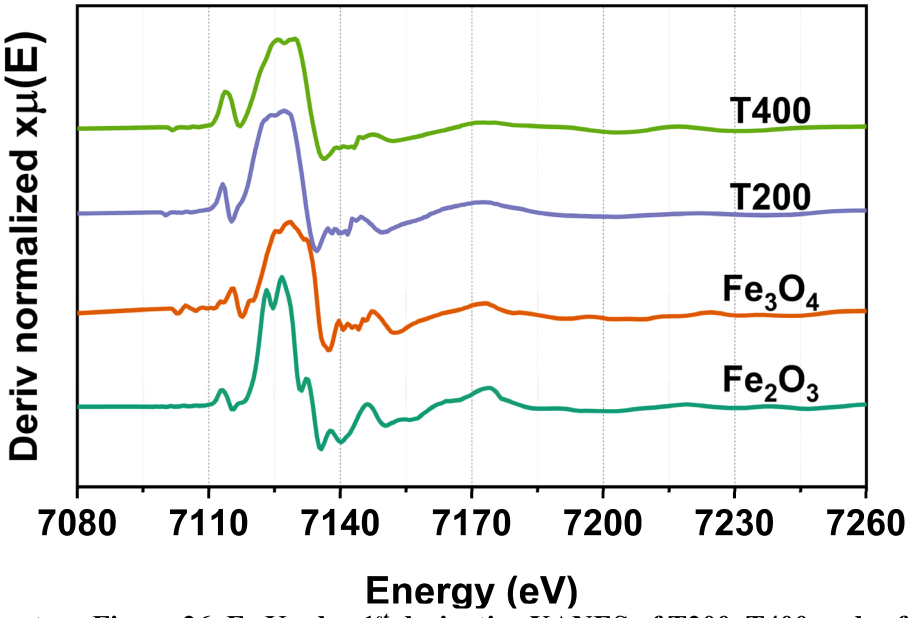

Supplementary Figure 26. Fe K-edge 1st derivative XANES of T200, T400 and references.
Source data are provided as a Source Data file.

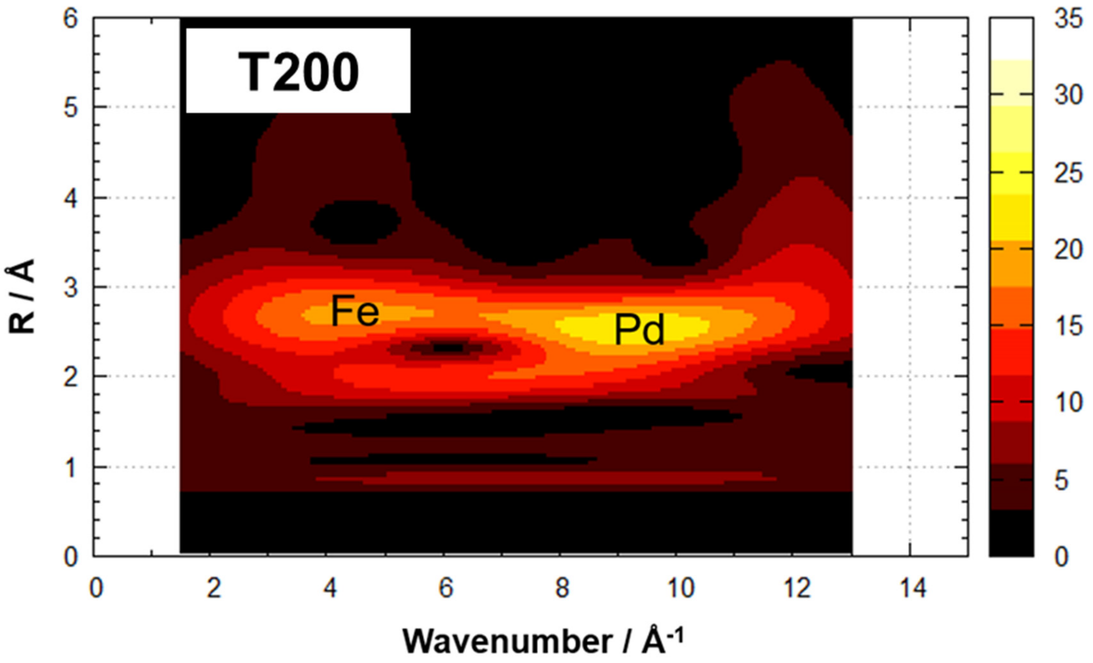

Supplementary Figure 27. WT EXAFS of T200.

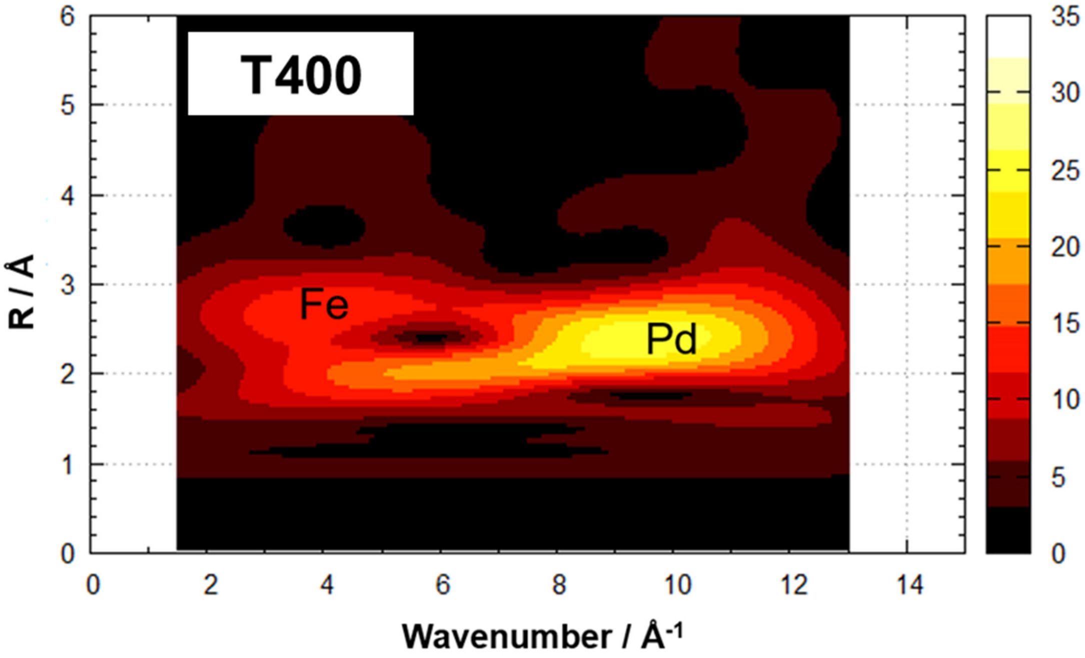

Supplementary Figure 28. WT EXAFS of T400.

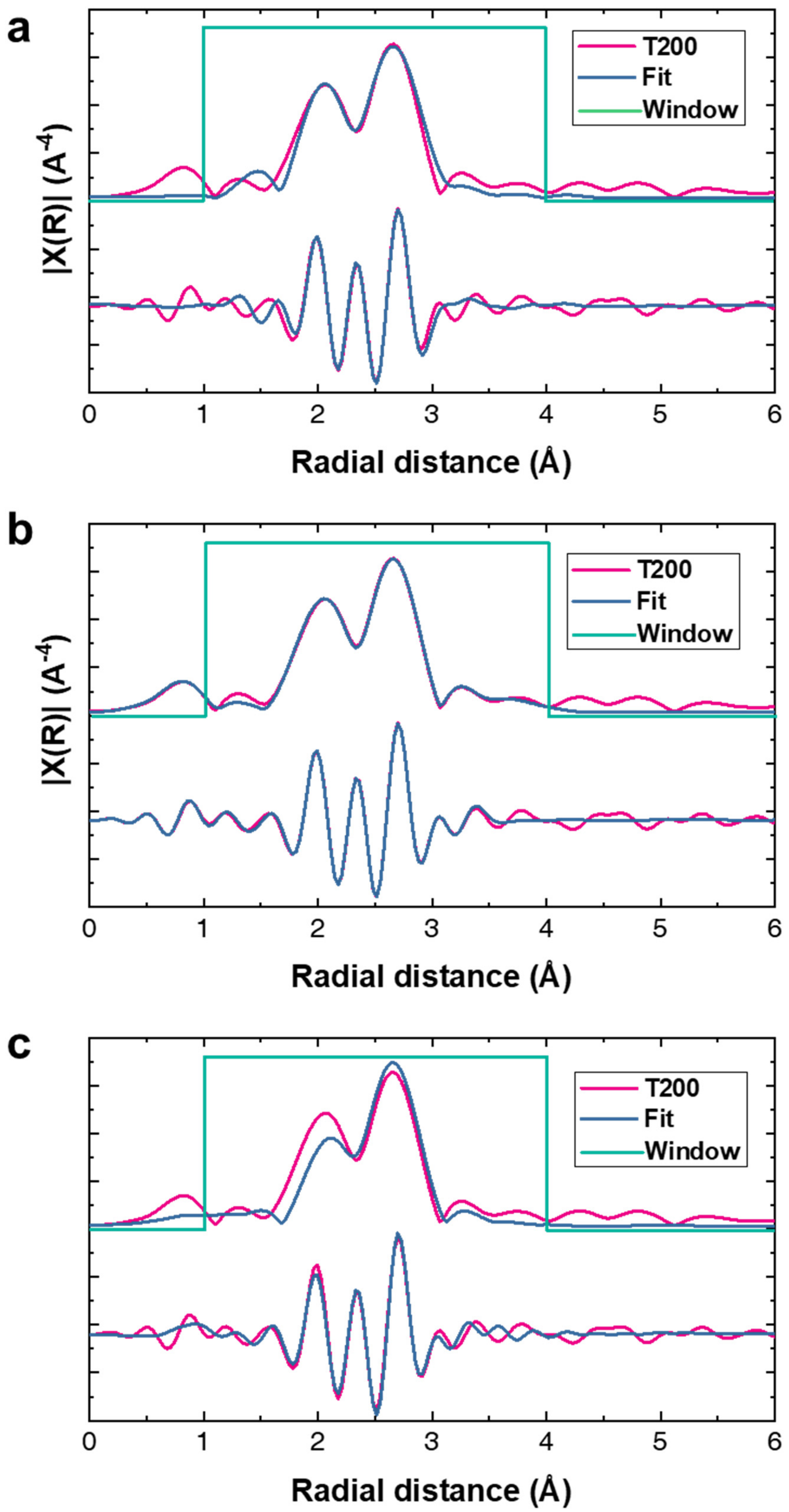

Supplementary Figure 29. Pd K-edge EXAFS fitting of T200. a) Pd on Fe_2O_3 surface; b) Pd on Fe_3O_4 surface; and c) Pd in Fe_3O_4 surface. Source data are provided as a Source Data file.

Supplementary Table 11. Curve-fitting results of Pd K-edge EXAFS spectra for T200 using simulated DFT structure model (ΔR = effective bond distance difference, σ^2 = mean-squared relative displacement, parentheses = error)

Simulated Model	Pd on Fe_2O_3	Pd on Fe_3O_4	Pd in Fe_3O_4
E_0	5.02 (0.12)	3.12 (0.42)	3.24 (0.38)
S_0^2	1.24	1.98	1.54
R-factor	0.045	0.021	0.051
ΔR_1	-0.003(0.008)	-0.001(0.003)	-0.006(0.001)
σ_1^2	0.010(0.0002)	0.003(0.005)	0.004(0.005)
ΔR_2	0.012(0.024)	-0.003(0.008)	0.005(0.002)
σ_2^2	0.010(0.021)	0.001(0.002)	0.010(0.024)
ΔR_3	-0.003(0.008)	-0.01(0.012)	0.021(0.042)
σ_3^2	0.087(0.001)	0.003(0.010)	0.003(0.001)
ΔR_4	0.016(0.012)	0.074(0.014)	-0.003(0.008)
σ_4^2	0.075(0.014)	0.003(0.001)	0.009(0.005)
ΔR_5	-0.07(0.014)	-0.012(0.005)	-0.003(0.008)
σ_5^2	0.008(0.005)	0.007(0.001)	-0.005(0.001)

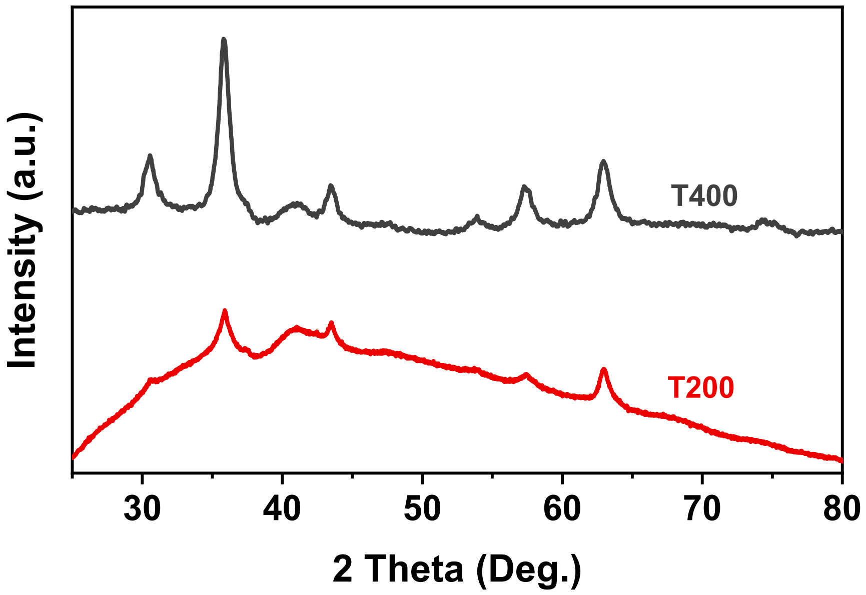

Supplementary Figure 30. XRD patterns of T200 and T400. Source data are provided as a Source Data file.

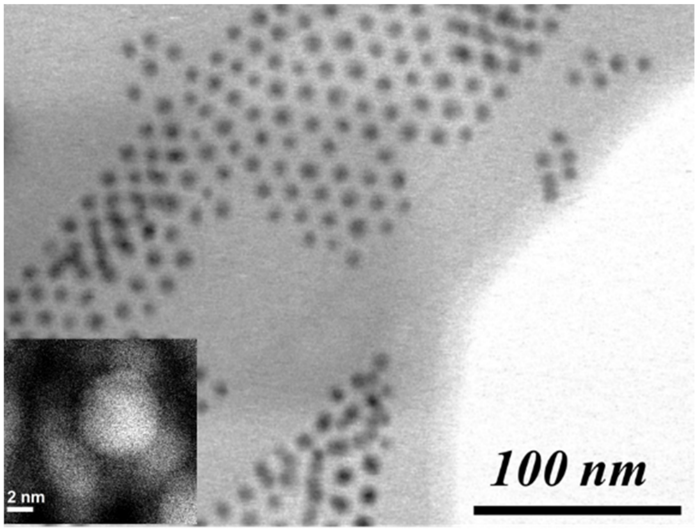

Supplementary Figure 31. TEM and HAADF-STEM (inset) images of the prepared ST1 NPs.

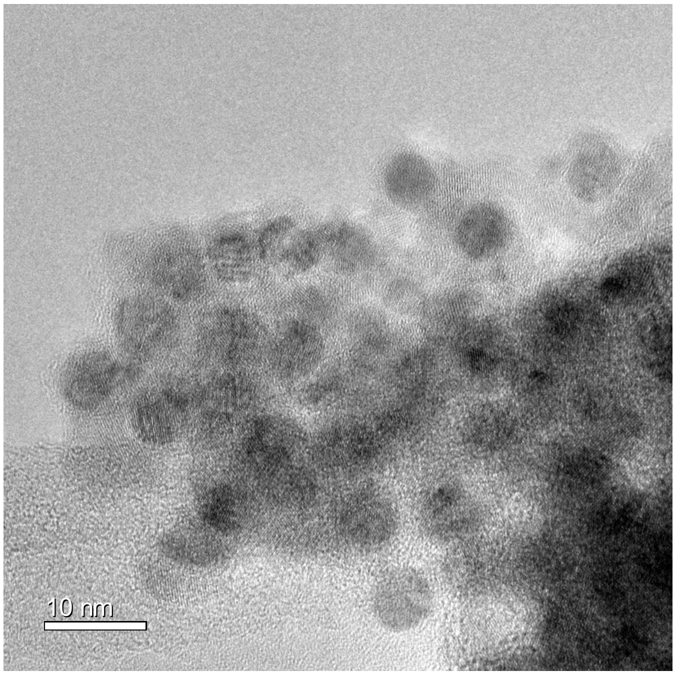

Supplementary Figure 32. The HAABF-STEM image of the prepared ST1.

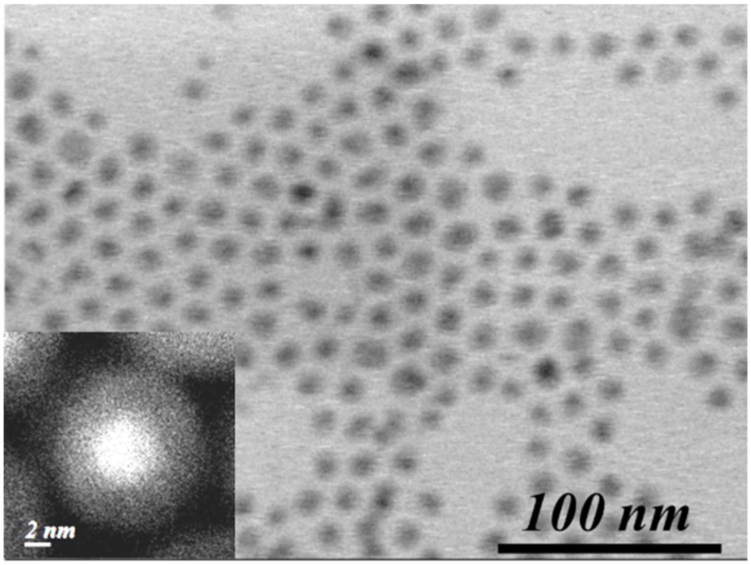

Supplementary Figure 33. TEM and HAADF-STEM (inset) images of the prepared ST3 NPs.

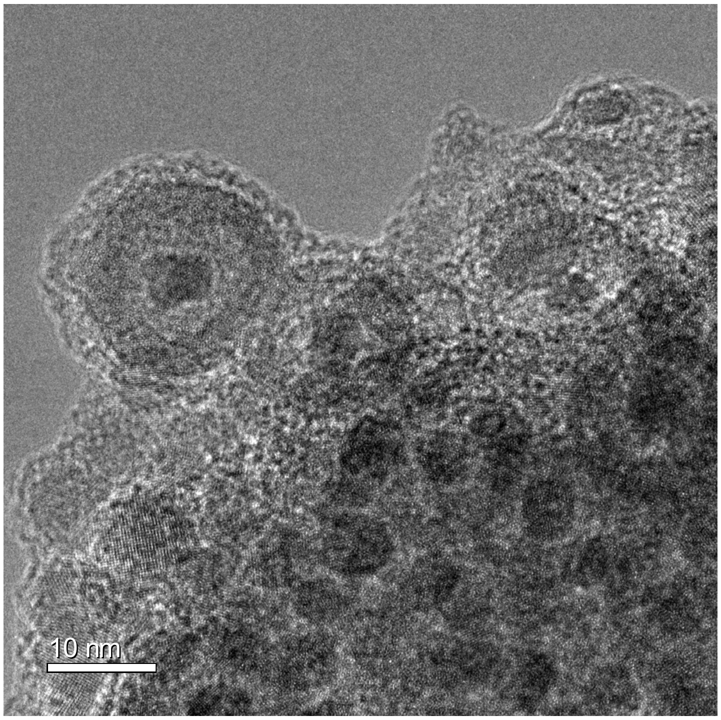

Supplementary Figure 34. The HAABF-STEM image of the prepared ST3.

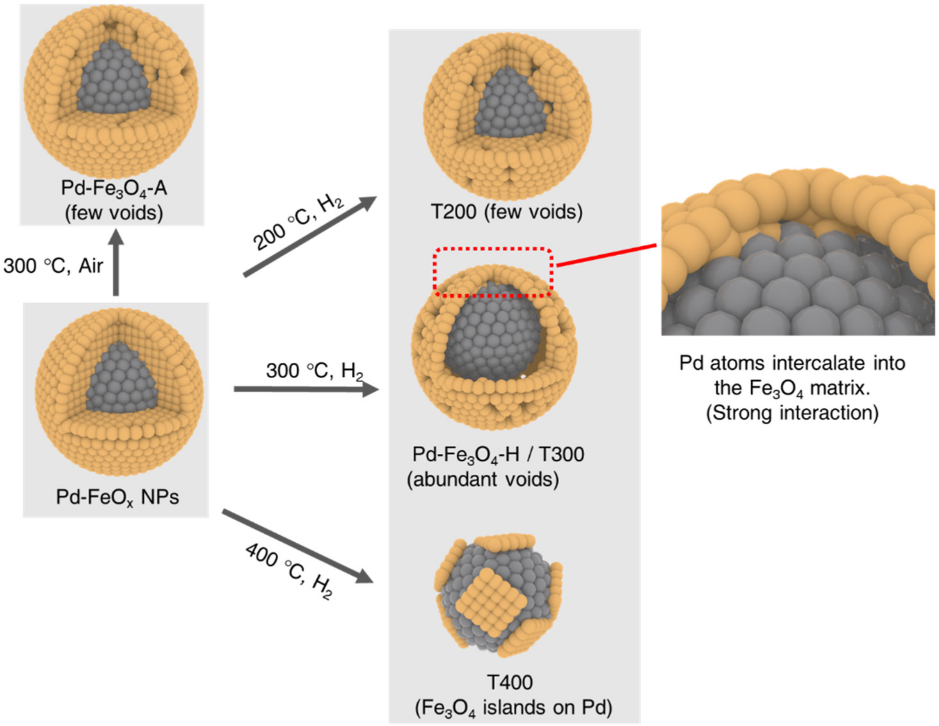

Supplementary Figure 35. Schematic diagram of samples obtained under different conditions.

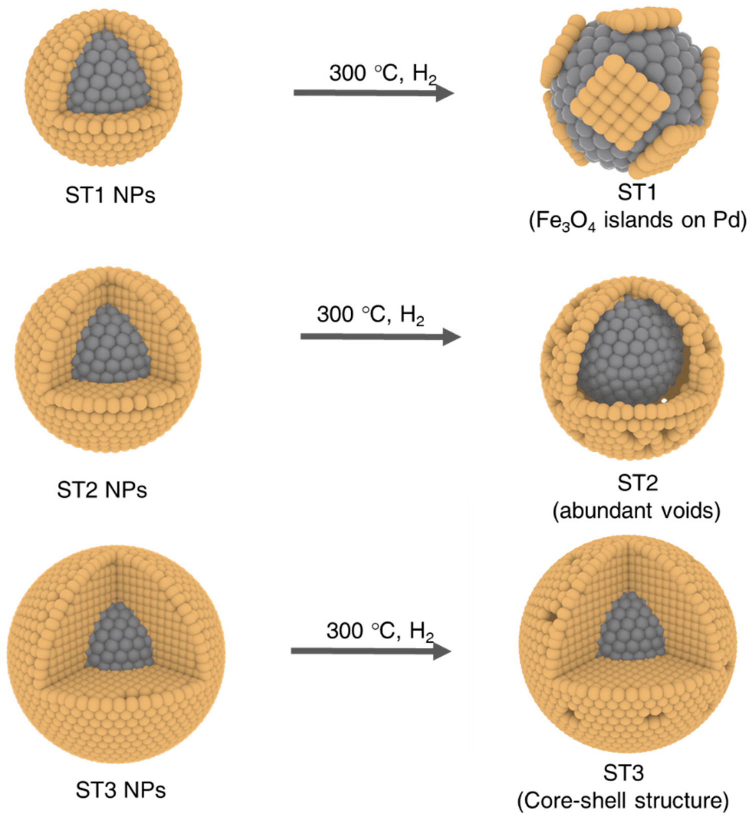

Supplementary Figure 36. Schematic diagram of samples with different thicknesses.

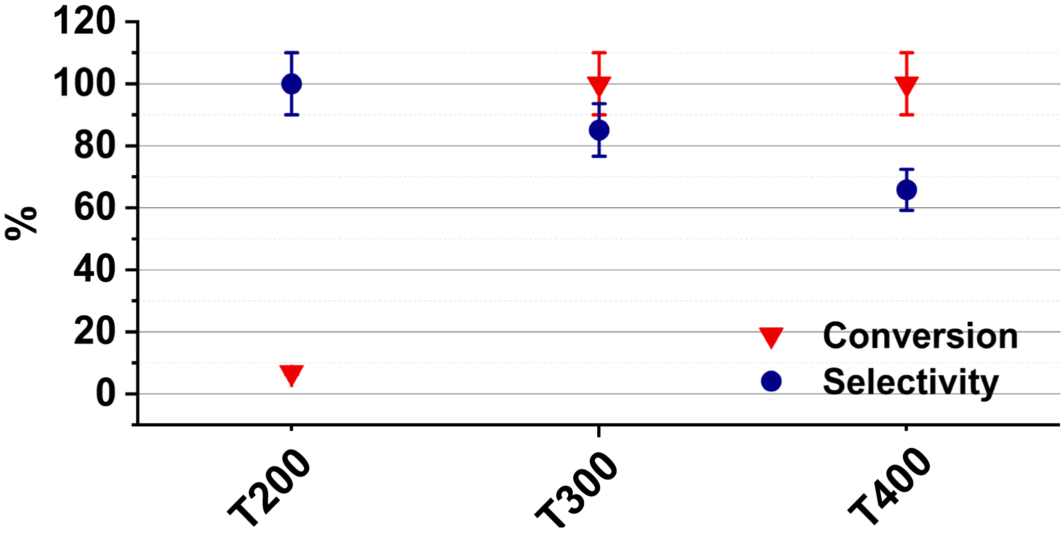

Supplementary Figure 37. Investigation of the effect of treating temperatures over the catalytic acetylene hydrogenation performance.

Reaction conditions: *m* (catalyst) = 15 mg; *v* (gas) = 50 sccm (0.6 sccm C_2H_2, 3 sccm H_2, 46.4 sccm He); *T* = 80 °C. Error bars represent the instrumental error (± 10 %). Source data are provided as a Source Data file.

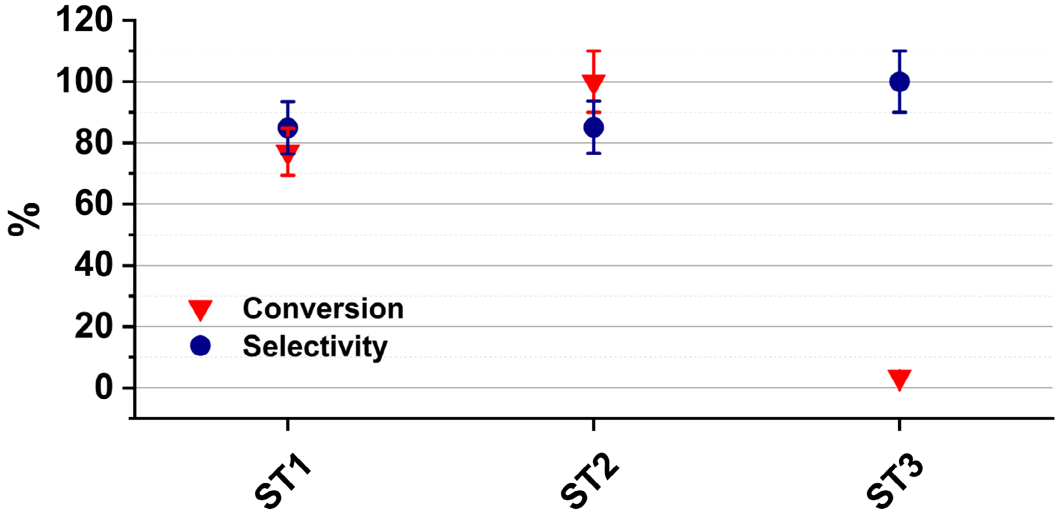

Supplementary Figure 38. Influence of the thickness of iron oxide shells over the catalytic acetylene hydrogenation performance.

Reaction conditions: *m* (catalyst) = 15 mg; *v* (gas) = 50 sccm (0.6 sccm C_2H_2, 3 sccm H_2, 46.4 sccm He); *T* = 80 °C. Error bars represent the instrumental error (± 10 %). Source data are provided as a Source Data file.

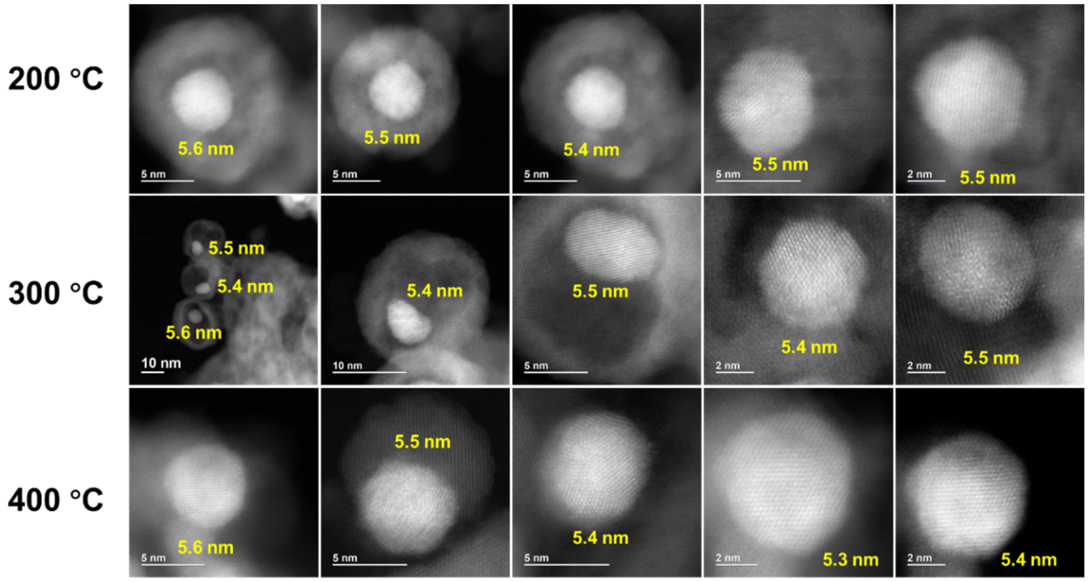

Supplementary Figure 39. HR-STEM images of Pd-Fe_3O_4 obtained at different temperatures.

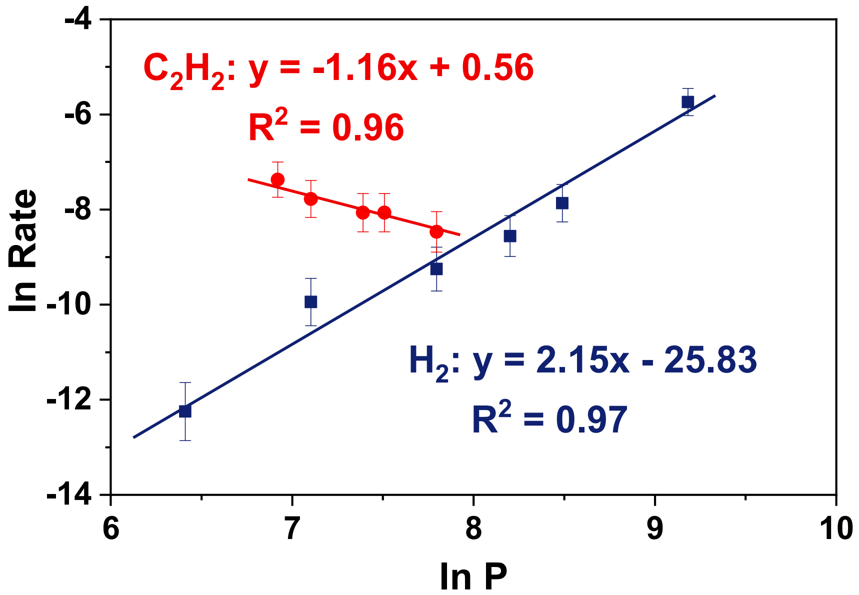

Supplementary Figure 40. Reaction order of the catalytic system.
Testing conditions: C_2H_2 order measurement: Pd-Fe_3O_4-H; *m* (catalyst) = 30 mg; *v* (gas) = 50 sccm (C_2H_2 is 0.5, 0.6, 0.8, 0.9, 1.2 sccm, H_2 is 5 sccm, balanced with He); *T* = 50 °C. H_2 order measurement: Pd-Fe_3O_4-H; *m* (catalyst) = 30 mg; *v* (gas) = 50 sccm (H_2 is 0.3, 0.6, 1.2, 1.8, 2.4, 4.8 sccm, C_2H_2 is 0.6 sccm, balanced with He); *T* = 50 °C. Error bars represent the instrumental error(± 10 %). Source data are provided as a Source Data file.

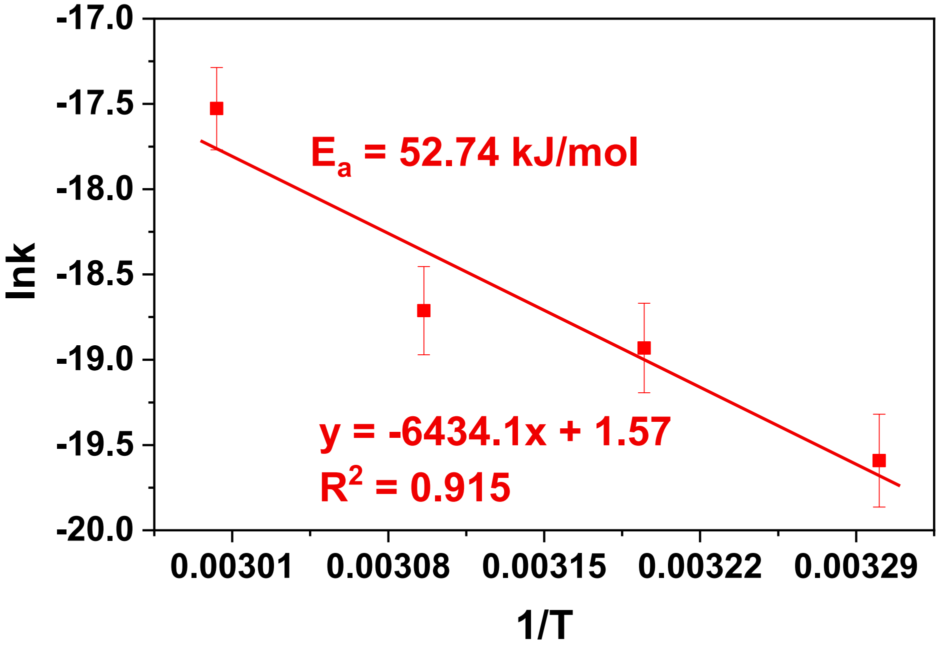

Supplementary Figure 41. Activation energy of the Pd-Fe_3O_4-H catalyst.
Reaction conditions: *m* (catalyst) = 30 mg; *v* (gas) = 50 sccm (C_2H_2 is 0.6 sccm, H_2 is 3 sccm, balanced with He); *T* = 30/40/50/60 °C. Error bars represent the instrumental error (± 10 %). Source data are provided as a Source Data file.

Supplementary References

1 Wei, S. *et al.* Direct Observation of Noble Metal Nanoparticles Transforming to Thermally S table Single Atoms. *Nat. Nanotechnol.* **13**, 856-861 (2018).

2 Vile, G. *et al.* A Stable Single-Site Palladium Catalyst for Hydrogenations. *Angew. Chem. Int. Ed.* **54**, 11265-11269 (2015).

3 Huang, F. *et al.* Atomically Dispersed Pd on Nanodiamond/Graphene Hybrid for Selective Hydrogenation of Acetylene. *J. Am. Chem. Soc.* **140**, 13142-13146 (2018).
